# Structural basis of KdpD histidine kinase binding to the second messenger c-di-AMP

**DOI:** 10.1016/j.jbc.2021.100771

**Published:** 2021-05-11

**Authors:** Anirudha Dutta, Mona Batish, Vijay Parashar

**Affiliations:** Department of Medical and Molecular Sciences, University of Delaware, Newark, Delaware, USA

**Keywords:** bacterial signal transduction, second messenger, c-di-AMP, osmoregulation, crystal structure, histidine kinase, methicillin-resistant *Staphylococcus aureus*

## Abstract

The KdpDE two-component system regulates potassium homeostasis and virulence in various bacterial species. The KdpD histidine kinases (HK) of this system contain a universal stress protein (USP) domain which binds to the second messenger cyclic-di-adenosine monophosphate (c-di-AMP) for regulating transcriptional output from this two-component system in Firmicutes such as *Staphylococcus aureus*. However, the structural basis of c-di-AMP specificity within the KdpD-USP domain is not well understood. Here, we resolved a 2.3 Å crystal structure of the *S. aureus* KdpD-USP domain (USP_Sa_) complexed with c-di-AMP. Binding affinity analyses of USP_Sa_ mutants targeting the observed USP_Sa_:c-di-AMP structural interface enabled the identification of the sequence residues that are required for c-di-AMP specificity. Based on the conservation of these residues in other Firmicutes, we identified the binding motif, (A/G/C)XSXSX_2_N(Y/F), which allowed us to predict c-di-AMP binding in other KdpD HKs. Furthermore, we found that the USP_Sa_ domain contains structural features distinct from the canonical standalone USPs that bind ATP as a preferred ligand. These features include inward-facing conformations of its β1-α1 and β4-α4 loops, a short α2 helix, the absence of a triphosphate-binding Walker A motif, and a unique dual phospho-ligand binding mode. It is therefore likely that USP_Sa_-like domains in KdpD HKs represent a novel subfamily of the USPs.

Nucleotide-based second messenger systems are signaling systems used by many bacteria and archaea to sense and respond to environmental signals (1). These systems metabolize various nucleotides in response to environmental cues (2), and specific interactions of these nucleotides with various protein and RNA receptors manifest physiological responses (2, 3, 4, 5). A recently discovered bacterial cyclic di-nucleotide second messenger, known as cyclic-di-adenosine monophosphate (c-di-AMP), is associated with antibiotic resistance, K^+^ homeostasis, DNA damage repair, virulence, sporulation, and day-night switches (6, 7, 8, 9, 10, 11, 12, 13, 14, 15, 16, 17).

Survival of almost all bacteria (other than gammaproteobacteria), and a few archaea, requires a minimum intracellular concentration of c-di-AMP (9, 12, 18, 19, 20). The binding of c-di-AMP to numerous receptor proteins essential for maintaining the homeostasis of required osmolytes, such as potassium and carnitine, regulates cellular integrity (13, 15, 20, 21, 22). By contrast, increased cellular accumulation of c-di-AMP also causes deleterious effects on the cells (21, 23, 24). Hence, c-di-AMP has been referred to as an “essential poison,” whose levels are regulated by the opposing activities of diadenylate cyclases and phosphodiesterases (13, 19, 25, 26, 27, 28).

Two-component systems (TCSs) are the other prevalent signaling systems in bacteria that sense and adaptively respond to environmental cues, utilizing mechanisms distinct from the second messenger systems (29, 30). In a prototypical TCS, an environmental signal is sensed by an extracellular domain of a membrane-bound histidine kinase (HK), which triggers its autophosphorylation. The HK then transfers the phosphoryl group to a response regulator protein, which then causes an intracellular response by altering gene expression (29, 30, 31, 32). While phosphotransfer mechanisms of TCSs have been extensively studied, the underlying principles of signal perception by HKs are not understood (33).

The KdpDE TCS is widespread in bacteria and archaea (34). It controls potassium homeostasis and virulence by regulating the transcription of multiple genes, including a kdpFABC operon, which encodes a high-affinity P-type ATPase transporter (35, 36, 37). The KdpD HKs of this TCS are prototypical among membrane-anchored HKs that contain an N-terminal sensory cytoplasmic region (NTR) in addition to a canonical transmembrane domain and a cytoplasmic C-terminal region. The C-terminal region contains a transmitter GAF domain and an EnvZ-like catalytic HK domain. The NTR is composed of KdpD' and universal stress protein (USP) domains (Fig. 1*A*) (38).

**Figure 1:**
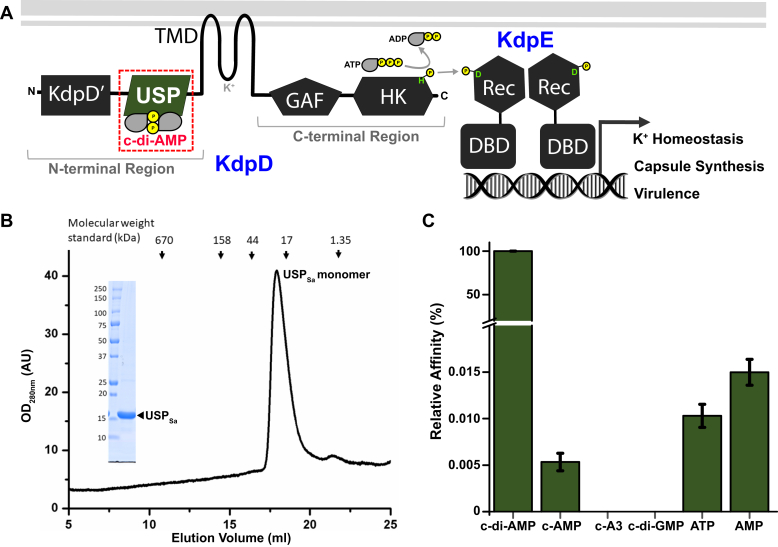
**Function, purification, and nucleotide preference of the USP sensory domain in *Staphylococcus aureus* histidine kinase KdpD.***A*, modular domain organization of KdpD (Pfam protein database accession number PF02702) in the KdpDE two-component system. The histidine kinase domain of KdpD acts as a kinase and a phosphatase to the response regulator transcription factor KdpE, and regulates >100 genes governing potassium homeostasis, capsule synthesis, and virulence in *S. aureus* (35). The structures of the canonical transmembrane domain (41), the GAF domain (42), the KdpD domain (Protein Data Bank ID: 2R8R), and the KdpE-bound histidine kinase domains (43) are known. The structure of the USP domain in the N-terminal region complexed to c-di-AMP (highlighted by a *box with a red dotted border*) has been determined in this study. *B*, analytical gel filtration chromatography of the purified USP_Sa_ domain from *S. aureus* KdpD (Pfam protein database accession number CAG41147, residues 213–364, MW_theor_:17.62 kDa) shows that USP_Sa_ forms monomers (MW_exper_:17.16 kDa) in solution. *Vertical arrows* above the absorbance trace indicate the peak positions of the gel filtration standards. The sodium dodecyl sulfate polyacrylamide gel electrophoresis picture shows the purity of USP_Sa_ after gel filtration. *C*, the nucleotide preference of USP_Sa_ was determined by an initial fluorescence change-based binding affinity analysis determined by MicroScale Thermophoresis (see Table S1 for dissociation constants). c-di-AMP, cyclic-di-adenosine monophosphate; GAF, cGMP-specific phosphodiesterases, adenylyl cyclases, and bacterial transcription factor FhlA domain; USP, universal stress protein; USP_Sa_, *S. aureus* KdpD-USP.

The mechanisms regulating transcriptional output from the KdpDE system have been well-studied in proteobacteria (34). The sensing of small molecule signals by different *Escherichia coli* KdpD HK (KdpD_*Ec*_) domains regulates its kinase and phosphatase activities (34). For example, the canonical transmembrane domain and the cytoplasmic C-terminal domain in KdpD_*Ec*_ sense the external and internal K^+^ concentrations, respectively (38), and the KdpD' domain in the NTR senses ATP (39). Also, the KdpD_Ec_ NTR is known to scaffold KdpE_Ec_ and the target DNA to further enhance the transcriptional output of the *E. coli* KdpDE system (KdpDE_Ec_) (40). While the structures of rest of the domains in KdpD are known (Fig. 1*A*) (41, 42, 43), the lack of knowledge concerning the three-dimensional structure of the USP in KdpD-NTR (USP_KdpD_) limits our understanding of its function. USP_KdpD_ belongs to a more widespread USP family of proteins (∼15 kDa), with members ubiquitously produced by all kingdoms of life (44). Generally, organisms possess multiple USP paralogs, most of which are single-domain proteins (hereafter referred to as *standalone USPs*), though a few (like USP_KdpD_) are parts of multidomain proteins (45, 46). Standalone USPs are essential for survival under stress conditions, such as high temperature, nutrition deficiency, oxidative stress, DNA damage, and other hostile environmental conditions (46, 47, 48, 49, 50). Based on their biochemical properties, standalone USPs are distributed among two subfamilies: (i) the USP_FG_ subfamily of proteins that bind ATP, and in some cases, hydrolyze it; and (ii) the USP_A_ subfamily of proteins that do not bind ATP (45, 51, 52). The structural determinants of ATP specificity in the USP_FG_ subfamily of proteins are well characterized (46, 53). In particular, a conserved Walker A motif G-X2-G-X9-G-(S/T) in USP_FG_ proteins mediates interactions between ribose and the phosphate moieties of ATP (46, 54, 55). However, USP_KdpD_ domains lack this motif, and they do not bind ATP as their preferred ligand (56).

In a high-throughput screen for c-di-AMP receptors in *Staphylococcus aureus*, the KdpD HK (hereafter referred to as KdpD_Sa_) initially emerged as the first HK receptor of c-di-AMP (7). Later, the KdpD HKs from *Listeria monocytogenes* (57) and *Synechococcus elongatus* (14) were also shown to bind c-di-AMP *in vitro*. Furthermore, the site of c-di-AMP binding in KdpD_Sa_ was found to be the USP domain in its NTR (hereafter referred to as USP_Sa_) (56). However, despite the importance of USP_KdpD_ in the KdpD function, the mechanisms underlying the mode and specificity of c-di-AMP binding to USP domains in KdpD homologs from Firmicutes (hereafter referred to as KdpD_Firmicutes_) has remained elusive because of the lack of structural information concerning the USP_KdpD_ domains (35, 38, 39, 56, 58). Here, we performed a structural and biochemical analysis of c-di-AMP-USP_Sa_ interactions, and we identified key c-di-AMP-binding residues in KdpD_Firmicutes_. Our studies identified a unique nucleotide-binding mode in USP_Sa_, intermediate between USP_FG_ and other nucleotide-binding proteins.

## Results

### USP_Sa_ specifically binds to c-di-AMP

Our initial attempts to express N-terminally hexahistidine (His_6_)-tagged USP_Sa_ in *E. coli* yielded an insoluble protein. Because the NTR of KdpD_Ec_ had previously been shown to scaffold KdpD, KdpE, and DNA (40), we reasoned that USP_Sa_ from the KdpD_Sa_-NTR might need KdpE_Sa_ for stability *in vivo*. Indeed, co-expression of the *S. aureus kdpE* gene (accession number SAR2167) with *usp*_*Sa*_ in *E. coli* produced soluble preparations of His_6_-tagged USP_Sa_, as well as untagged USP_Sa_ (Fig. 1*B*).

To determine the binding affinity and specificity of this purified His_6_-USP_Sa_ protein toward c-di-AMP, we monitored the ligand-induced fluorescence change using MicroScale Thermophoresis (MST). In this assay, His_6_-USP_Sa_ bound to c-di-AMP with a dissociation constant (K_D_) of 0.5 ± 0.06 μM (Table S1), which is consistent with a previously reported K_D_ of 2 ± 0.18 μM using a maltose-binding protein–tagged USP in a differential radial capillary action of ligand assay (56). Furthermore, the binding of His_6_-USP_Sa_ was highly specific to c-di-AMP, because the dissociation constants for other second messengers (cAMP, c-tri-AMP, and c-di-GMP) were significantly higher than for c-di-AMP (Fig. 1*C* and Table S1). The binding affinity of His_6_-USP_Sa_ to AMP and ATP (the precursor of c-di-AMP) was more than ∼5000-fold lower than that of c-di-AMP. To determine whether the binding of c-di-AMP stabilizes USP_Sa_, we employed a fluorescence-based thermal shift (ThermoFluor) assay (59, 60), and we found that the addition of a 7.5-fold molar excess of c-di-AMP to purified untagged USP_Sa_ increases its melting temperature by 10 °C (Fig. S1*D*). These data support the view that USP_Sa_ is the founding member of a new branch of USP-family proteins that utilize c-di-AMP as their preferred nucleotide ligand (56).

### Overall structure of USP_Sa_ bound to c-di-AMP

**Figure 2:**
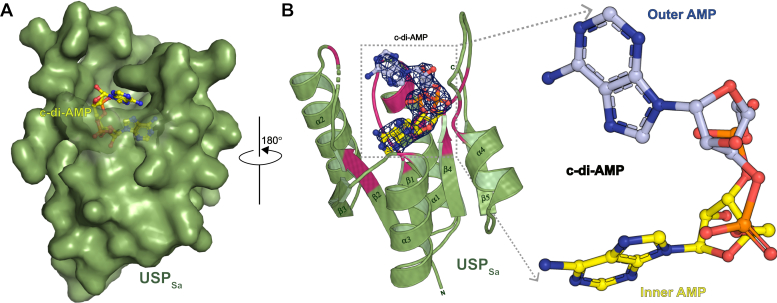
**X-ray crystal structure of USP**_**Sa**_**:c-di-AMP at 2.3 Å.***A*, the X-ray crystal structure of USP_Sa_ (green surface) complexed with c-di-AMP (*ball-and-stick model*). *B*, the X-ray crystal structure of USP_Sa_ (*green cartoon*) complexed with c-di-AMP (*ball-and-stick model*). Composite electron density map (2F_o_-F_c_, contoured at 2.0 σ) of c-di-AMP in the refined USP_Sa_:c-di-AMP structure is shown as a blue mesh. The USP_Sa_ residues at the USP_Sa_:c-di-AMP structural interface are colored *magenta*. To obtain this view of the USP_Sa_:c-di-AMP model, the structure illustrated in panel A was rotated 180° in the direction indicated by the *arrow*. β2 is connected to α2 by a flexible linker, shown as a *dashed green line*. The zoomed-in inset on the right shows an expanded view of c-di-AMP (ball-and-stick model), highlighting the “outer” carbon atoms of the AMP moiety in *light blue,* and highlighting the “inner” carbon atoms of the AMP moiety in *yellow*. This c-di-AMP coloring scheme is utilized throughout the text. c-di-AMP, cyclic-di-adenosine monophosphate; USP, universal stress protein; USP_Sa_, *S. aureus* KdpD-USP.

We determined the USP_Sa_:c-di-AMP complex structure at 2.3 Å resolution using X-ray crystallography (Table 1). The asymmetric unit of the structure comprises of a single USP_Sa_ molecule (Fig. 2*A*). USP_Sa_ contains four repeats of alternating β-strand-α-helix motifs (β-α)_4_, followed by a fifth β-strand-α-helix motif (β_5_) (Fig. 2*B*). A sheet of five β-strands (β1-β5) in the structure is sandwiched between two layers of α-helices, with each layer containing two α-helices (α1-α2 and α3-α4) (Fig. 2*B*). There was no interpretable electron density for the N-terminal residues T213-L235 and for the β2-α2 loop residues K277-S279, indicating that there is high structural flexibility in these regions.

**Table 1:** Structural data collection and refinement statistics

Data	USP_Sa_-CDA	Se-USP_Sa_-CDA
Protein Data Bank ID	7JI4	
Data collection		
Space group	P4_3_2_1_2	P4_3_2_1_2
Cell dimensions		
a, b, c (Å)	54.41, 54.41, 96.53	54.56, 54.56, 97.50
α = β = γ (°)	90	90
Wavelength (Å)	0.9774	0.9791
Resolution (Å)	47.4 (2.382–2.300)	38.58 (2.54–2.51)
R_merge_	11.2 (44.4)	9.7 (34.5)
Average I/σI	33.8 (7.0)	22.1 (7.5)
Completeness (%)	99.8 (99.1)	98.0 (97.7)
Multiplicity	22.9 (22.6)	11.5 (11.7)
Total reflections	157,974 (15,107)	79,606 (7814)
Unique reflections	6909 (662)	5444 (603)
CC_1/2_	99.4 (98.2)	98.9 (98.7)
Solvent content (%)	41.2	42.1
SAD phasing		
Figure of merit		0.33
Refinement		
R_work_/R_free_	20.77/24.90	
RMS deviations		
Bond lengths (Å)	0.008	
Bond angles (°)	1.049	
Number of atoms		
All atoms	1030	
Protein	962	
Ligand	44	
Water	24	
Ramachandran statistics		
Favorable (%)	99.17	
Additionally allowed (%)	0.83	
Outlier (%)	0	

CDA, 3′, 5′-cyclic di-adenosine monophosphate (c-di-AMP); SAD, single-wavelength anomalous dispersion; USP, universal stress protein; USP_Sa_, *S. aureus* KdpD-USP.

Rwork  =  Σ ∥Fo|−|Fc∥/Σ |Fo|, calculated with a working set of reflections. Rfree is Rwork calculated with only the test set with 10% of reflections. Data for the highest resolution shell are given in parentheses. The structures were determined using single crystals. The reflections I(+) and I(−), related by Friedel's Law, were treated as independent for the purpose of the SAD data only.

Multiple rounds of model-building and structural refinement unveiled a readily interpretable electron density corresponding to c-di-AMP in the difference-density F_O_-F_C_ Fourier map. The c-di-AMP was modeled after the USP_Sa_ model was nearly complete. Further refinement highlighted the excellent agreement between the c-di-AMP model and the difference-density map (Fig. 2*B*). The c-di-AMP binding region in the USP_Sa_ was found to involve β1, α1, the β1-α1 loop, β2, the β2-α2 loop, β4, and the β4-α4 loop (Fig. 2*B*). One of the two AMP moieties of the c-di-AMP in the USP_Sa_:c-di-AMP structure occupies a pocket similar to the ATP-binding pocket in the USP_FG_ subfamily of proteins, which we refer to as the “inner AMP” (see the Experimental procedures section, Fig. 2*B* inset, and Fig. 4, *A* and *B*). The other AMP moiety of c-di-AMP in the USP_Sa_:c-di-AMP structure projects outward from the “ATP-binding pocket”, and we refer to it as the “outer AMP” (Fig. 2*B* inset, and Fig. 4, *A* and *B*). Similarly, we refer to the adenine, ribose, and phosphoryl groups belonging to the inner and outer AMP in c-di-AMP as the “inner” and “outer” adenine, ribose, and phosphoryl groups, respectively.

### Conformation adopted by c-di-AMP in the USP_Sa_:c-di-AMP structure

**Figure 3:**
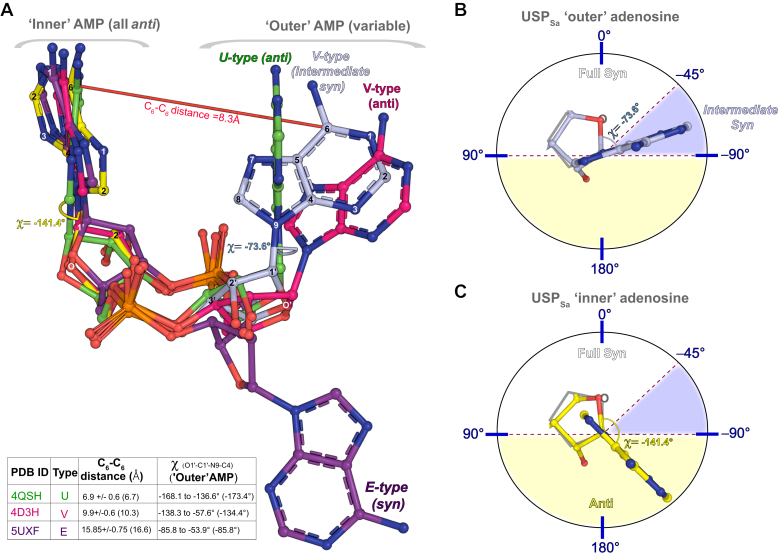
**The outer AMP in the USP**_**Sa**_**:c-di-AMP structure adopts a V-type *Intermediate Syn* conformation.***A*, structural superposition of a c-di-AMP model (shown as a ball-and-stick representation possessing *light blue* “outer” and *yellow* “inner” AMP moieties) from the USP_Sa_:c-di-AMP structure, in combination with different known c-di-AMP models that form either a U-type conformation (*green*, from PDB IDs 4QSH), a V-type conformation (*magenta*, from PDB IDs 4D3H), and an E-type (*purple*, from PDB IDs 5UXF) conformation (9). Also shown are the C_6_-C_6_ distance (Å) for two AMP moieties (*red line*), and the N-glycosidic torsional angles (χ, measured between the O_1_′-C_1_′-N_9_-C_4_ atoms, determined by the PyMOL molecular visualization system) of the outer and inner adenosines in the USP_Sa_:c-di-AMP structure (92). The inset table summarizes the mean C_6_-C_6_ distances, including the standard deviation between the two AMP moieties (shown in Å in the third column), as well as the χ angles of the “outer” equivalent AMP moieties (shown in the fourth column) of all known U-type (from Protein Data Bank IDs: 4QSH, 4XTT, 4YP1, 5CFN, and 5F29), V-type (from Protein Data Bank IDs: 4RLE, 4RWW, 4WK1, 4D3H, and 4S1B), and E -type (from Protein Data Bank IDs: 5XSN, 5UXF, and 4QSH2) c-di-AMP structures. The C_6_-C_6_ distances and χ angles for the shown U-type, V-type, and E-type c-di-AMP models (Protein Data Bank IDs 4QSH, 4D3H, and 5UXF) are provided within the parentheses of the third and fourth columns, respectively. *B and C*, show the χ-angle-based conformational classification of the outer (*light blue ball-and-stick model*) and inner (*yellow ball-and-stick model*) adenosines from the USP_Sa_:c-di-AMP structure, in an *intermediate syn* conformation (adenine occurring in the li*ght blue section* of the figure), and in an *anti* conformation (adenine occurring in the *yellow section* of the figure). c-di-AMP, cyclic-di-adenosine monophosphate; USP, universal stress protein; USP_Sa_, *S. aureus* KdpD-USP.

Based on the relative distance and spatial arrangement of the AMP moieties in the c-di-AMP molecules, the receptor-bound c-di-AMP molecules are known to adopt four different conformations: U-, V-, E-, and O-types (Fig. 3*A*) (9). The two adenine bases of U-type c-di-AMP molecules form a parallel “stacking” arrangement with an average distance of 6.9 Å between their C_6_ atoms (Fig. 3*A*, green sticks). The adenine bases in the E-type and O-type adopt an antiparallel arrangement, with an average C_6_-C_6_ distance of 15.8 Å and 18.1 Å, respectively (Fig. 3*A*, purple sticks). V-type adenine bases, by contrast, adopt an intermediate unstacking arrangement, with an average C_6_-C_6_ distance of 9.9 Å (Fig. 3*A*) (9). Furthermore, based on the N-glycosidic torsional angle (χ) in each AMP, the adenine ring may adopt an *anti* conformation (if −90° ≥ χ ≥ 90°) or a *syn* conformation (if 90° ≥ χ ≥ −90°), where the latter can be further subcategorized into a *full syn* (if 90° ≥ χ ≥ −45°) or an *intermediate syn* (if −45° ≥ χ ≥ −90°) conformation (Fig. 3, *B* and *C*) (61). Both adenosine moieties in the known U-type, O-type, and V-type structures (except for a V-type c-di-AMP from Protein Data Bank ID 4RWW) adopt *anti* conformations, and one of the adenosine moieties in the E-type structures adopts an *intermediate syn* conformation (Fig. 3*A*, inset) (62). The c-di-AMP in the USP_Sa_:c-di-AMP structure adopts a V-shaped conformation with a C_6_-C_6_ distance of 8.3 Å (Fig. 3*A*). Further, the outer and inner AMPs of the c-di-AMP in the USP_Sa_:c-di-AMP structure are oriented in an *intermediate syn* conformation (χ = −73.6°) and in an *anti* conformation (χ = −141.4°), respectively (Fig. 3, *B* and *C*). While the exact functional relevance of this conformational diversity in c-di-AMP is unclear, it likely reflects the structural adaptability of this cyclic di-nucleotide that enables it to bind to pockets of different shapes (9, 63).

### USP_Sa_ and USP_Sa_:c-di-AMP complex exhibit unconventional monomeric forms

Most of the standalone USPs form crystallographic symmetry-related dimers called type-I dimers (46). The asymmetric unit of the USP_Sa_:c-di-AMP structure also exhibits a USP_Sa_ dimeric interface, with a symmetry-mate obtained by the operation X, Y, −Z + 1. Because this interface shows a decrease in solvent-accessible surface area of 867.9 Å^2^ per molecule, we analyzed the possibility that USP_Sa_ can make such dimers, utilizing analytical gel filtration. We found that USP_Sa_ in the absence of c-di-AMP exists predominantly in a monomeric form (Fig. 1*B*). Furthermore, sedimentation velocity analytical ultracentrifugation (SV-AUC) of USP_Sa_ in the absence of c-di-AMP (*apo*-form) and in the presence of an approximately two-fold excess of c-di-AMP was carried out (Fig. S1, *A–C*). While a minor population of the *apo*-form and c-di-AMP-bound USP_Sa_ exhibited sedimentation coefficients (*s*_20,w_) of 3.3S and 2.9S, a significant population exhibited *s*_20,w_ values of 1.9S and 1.8S, respectively (Table S2). Comparison of these results with the theoretically calculated *s*_20,w_ values of 1.7S and 2.6S for the monomeric and dimeric USP_Sa_ models obtained from the USP_Sa_:c-di-AMP structure (Table S2) suggests that the majority of the *apo*-form and c-di-AMP–bound USP_Sa_ preparations exist as monomers in solution. Our data, however, do not rule out the possibility of heterodimerization of USP_Sa_ with other standalone USPs in *S. aureus* (*e.g.,* ACOL1753 and SACOL1759). Such interactions between the USP domain in the *E. coli* KdpD (USP_Ec_) and the standalone USP-C protein have been reported to scaffold the KdpE_Ec–_DNA complex (58). Furthermore, SV-AUC analysis also suggested a possible conformational change underlying the transition between the *apo*-form and c-di-AMP-bound USP_Sa_ states (Fig. S1, *A–C*), which is consistent with the *in vitro* stabilization of USP_Sa_ in the presence of c-di-AMP, mentioned above (Fig. S1*D*).

### USP_Sa_ shares an inner adenosine binding mode with USP_FG_ proteins

**Figure 4:**
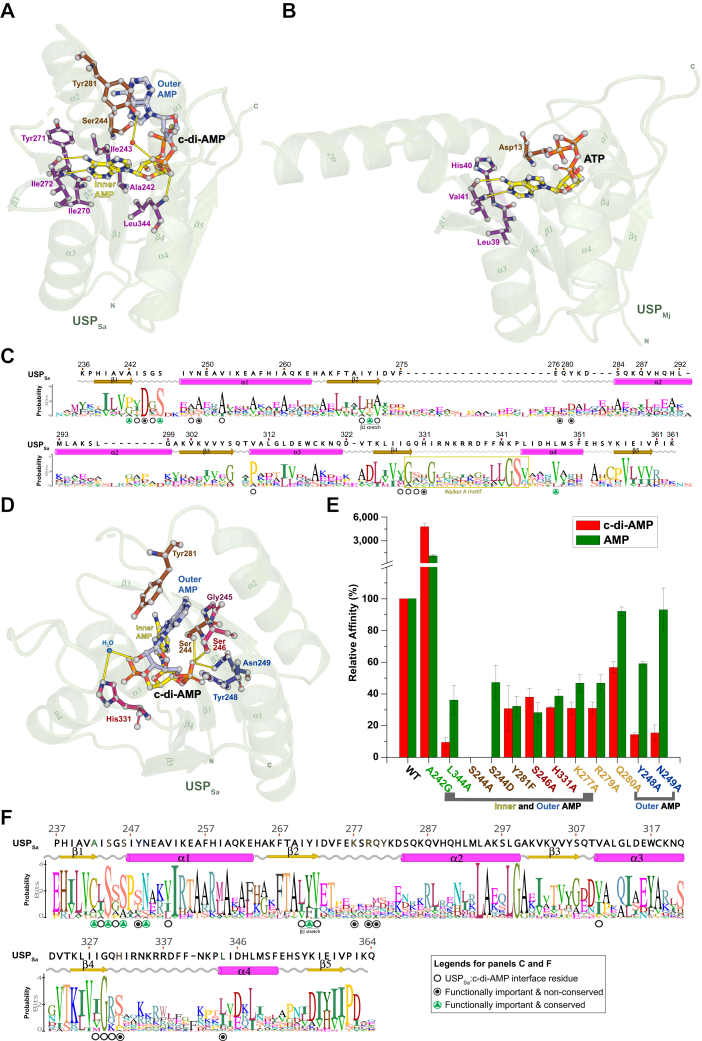
**Interactions of c-di-AMP with USP**_**Sa,**_**and comparison with other USP homologs.***A*–*C*, show interactions of the inner AMP in c-di-AMP with USP_Sa_, and a comparison with USP_FG_ proteins. *A*, the interactions of USP_Sa_ (*green transparent cartoon*) with the inner adenine of c-di-AMP (represented by *yellow balls and sticks*). The USP_Sa_ residues at the interface with the inner adenine of c-di-AMP are shown in a ball-and-stick representation with *gray balls*, and *magenta and brown sticks*. *B*, an ATP-bound USP_FG_ protein (USP_Mj_ from *Methanocaldococcus jannaschii*, PDB ID: 1mjh) is shown for comparison with the c-di-AMP-bound USP_Sa_ structure shown in panel A. All of the representations are similar to *panel A*, and they show conservation of adenine-binding residues from USP_Mj_ as *magenta sticks*. *C*, structure-guided sequence alignment of USP_ATP_ proteins (Protein Data Bank IDs 1MJH, 5AHW, 3S3T, 3FDX, 2JAX, and 3HGM) with USP_Sa,_ depicted in a sequence logo representation. *D*, interactions of the outer AMP (*light blue sticks*) in c-di-AMP with USP_Sa_ residues (*red, brown, and blue sticks*) in the USP_Sa_:c-di-AMP structure. Water molecules are depicted as *blue spheres*. To depict the orientation of the USP_Sa_ residues (shown as *balls and sticks*) in *panels A* and *D* relative to the rest of the USP_Sa_ structure, that structure is shown as a green transparent cartoon, with labeled secondary structure elements, as well as N termini and C termini. All c-di-AMP and USP_Sa_ atoms shown in the ball-and-stick representations in *panels A*, *B*, and *D* are colored *gray*, and the hydrogen bonds between atoms are depicted as *yellow lines*. *E*, relative binding affinities (%) of USP_Sa_ mutants targeting the USP_Sa_:c-di-AMP structural interface with c-di-AMP (*red bars*) and with AMP (*green bars*) (see methods for description, and Table S1 for dissociation constants). *Square brackets* at the bottom of the plot differentiate USP_Sa_ mutants with altered binding affinities for both c-di-AMP and AMP (*i.e.,* deficient in binding both the inner and the outer AMPs) from mutants with altered binding affinities for c-di-AMP only (*i.e.,* mutants deficient in binding the outer AMP in c-di-AMP). The *x*-axis labels identify mutants of USP_Sa_ residues that are present at the structural interface with the inner AMP (*green text*), at the structural interface with the outer AMP (*red or blue text*), and at the structural interfaces with both the inner and the outer AMPs (*brown text*), as well as identifying mutants possessing residues in an unbuilt region of USP_Sa_:c-di-AMP structure (*orange text*). *F*, sequence alignment of KdpD_CDA_ proteins (see Experimental procedures for description) depicted in a sequence logo representation. In both panels C and F, circles at the bottom of the alignments identify USP_Sa_ residues at the USP_Sa_:c-di-AMP structural interface. Circles filled with *green triangles* identify residues that are conserved among the homologs (>65% similarity) and that showed significant loss or gain of function in the c-di-AMP binding assay (see *panel C* and (56)). *Circles filled with black dots* identify residues that are not conserved but showed significant loss of function in the c-di-AMP binding assay. *Empty circles* identify USP_Sa_:c-di-AMP structural interface residues that were not subjected to mutagenesis and functional analysis in this study. The conservation of residues at each position is depicted by the size of the letters in the sequence logo, where the most conserved residues are highlighted by a larger sized letter. Logo letters that are colored *blue, green, red, and black* indicate basic, polar, acidic, and hydrophobic residues, respectively. The numbering of the residues is based on their positions within the USP_Sa_ domain of the *S. aureus* KdpD (accession number CAG41147). The secondary structure elements are derived from the USP_Sa_:c-di-AMP structure (in which α-helices are shown as *magenta cylinders*, and β-sheets are shown as *yellow arrows*). See Figure S2 for the full universal stress protein sequence alignments used to prepare these sequence logos. c-di-AMP, cyclic-di-adenosine monophosphate; USP, universal stress protein; USP_Sa_, *S. aureus* KdpD-USP.

The inner adenine of c-di-AMP occupies a pocket lined by the residues in the β2, β1, and β1-α1 loop in USP_Sa_ (Fig. 4*A*). A structural comparison of USP_Sa_ with USP_ATP_ proteins shows a shared adenine-binding mode, with many of the adenine interacting residues in this pocket being conserved (Fig. 4, *A* and *B*). In USP_ATP_ proteins, three consecutive residues at the end of β2 (hereafter called the “*β2 stretch*”, magenta colored residues in Fig. 4*B*) and a fully conserved Asp residue in the β1-α1 loop mediate these interactions (46). More specifically, the side chains of the residues at the first two positions in the *β2 stretch*, conserved as aliphatic (Val/Ile/Leu) and hydrophobic (Tyr/His/Thr) residues, and the side chain of a conserved Asp in the β1-α1 loop enable hydrophobic interactions with the adenine. The third *β2 stretch* residue is not conserved in USP_ATP_ proteins and uses main-chain carboxyl and amino groups to form hydrogen bonds with adenine N6 and N1 (Figs. 4, *B* and *C*, and S2*A*) (46). Similar to USP_ATP_ proteins, the side chains of the first two *β2 stretch* residues in USP_Sa_ (Ile270 and Tyr271) are able to form hydrophobic contacts with the inner adenine ring of c-di-AMP, and the main chain carboxyl and amino groups of the third residue (Ile272) form hydrogen bonds with adenine N6 and N1, respectively (Fig. 4*A*). While our attempts to obtain soluble preparations of the *β2 stretch* mutants (I270A, Y271A, and I272G) to analyze their c-di-AMP binding affinity failed, a maltose-binding protein–tagged version of the USP_Sa_ Y271A mutant has previously been shown to be deficient in c-di-AMP binding *in vitro* (56). Furthermore, KdpD_CDA_ proteins contain an aliphatic (Val/Ile/Leu) and hydrophobic (Tyr/Phe) residue at the first and second *β2 stretch* positions, respectively, which shows the importance of the *β2 stretch* residues for engagement of the inner adenine ring (Figs. 4*F* and S2*B*).

The last residue in the USP_Sa_ β1 (Ala242) also makes (i) side chain–mediated hydrophobic contacts with the inner adenine ring and (ii) a main chain–mediated hydrogen bond with the 2′-hydroxyl group of the inner ribose (Fig. 4*A*). Because KdpD_CDA_ and USP_ATP_ proteins contain a nonbulky residue (Cys/Ala/Ser/Gly) in KdpD_CDA_ and a (Pro/Val/Thr) in USP_ATP_ proteins at this position (Figs. 4, *C* and *F*, and S2) (46), we hypothesized that a nonbulky residue at this position would decrease steric repulsion to the inner adenine ring and therefore increase the flexibility of the main chain, thereby better accommodating the inner ribose. Indeed, mutation of Ala242 to a less bulky Gly residue (A242G) dramatically improved the binding affinities of USP_Sa_ for both c-di-AMP (∼50 fold) and AMP (∼25-fold) (Fig. 4*E* and Table S1). Therefore, we believe that the residues in the *β2 stretch* and the last residue of β1 comprise an adenosine-binding pocket that is conserved in all USP-family proteins and that KdpD_CDA_ proteins use this pocket to engage the inner AMP in c-di-AMP.

### USP_Sa_ exhibits a unique β4-α4 loop conformation

**Figure 5:**
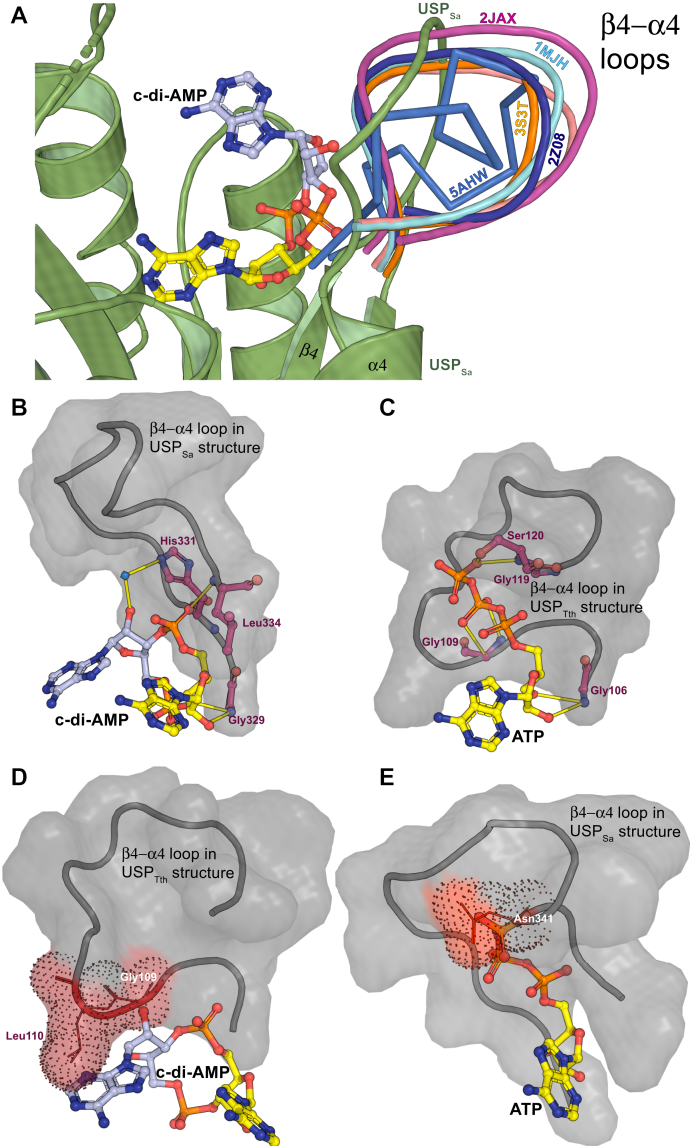
**Comparison of Walker loop conformations and ligand specificity in USPs.***A*, structural superposition of the USP_Sa_ (*green cartoon*) with ATP-bound and cAMP-bound standalone USP_FG_ proteins, showing that the USP_Sa_ β4-α4 loop adopts a distinct conformation (the color of each Protein Data Bank ID label matches the color of its loop; and the USP_Sa_ loop is *green*). *B*, conformation of the USP_Sa_ β4-α4 loop (shown as a *gray cartoon* with a transparent surface) showing the positions of Gly329, Leu334, and His331 (*balls and sticks colored magenta*) that enable direct or water-mediated hydrogen-bonding interactions (*yellow lines*) with c-di-AMP (*balls and sticks colored yellow and light blue*). *C*, conformation of the β4-α4 loop in an ATP-binding universal stress protein from *Thermus thermophilus* HB8 (USP_Tth_ from Protein Data Bank ID 2Z08, the closest USP_Sa_ structural homolog determined using the MADOKA protein similarity search (93)), shown as a *gray cartoon* with a transparent surface. USP_Tth_ positions the Walker motif residues Gly106, Gly109, Gly119, and Ser120 (balls and sticks colored *magenta*) that form hydrogen bonds (*yellow lines*) with the ribose and phosphoryl moieties of ATP (*balls and sticks colored yellow*). *D*, modeling of the c-di-AMP molecule (from the USP_Sa_:c-di-AMP structure) in the binding pocket of USP_Tth_ (superimposed onto the USP_Sa_ structure) shows that the outer AMP moiety of c-di-AMP would slightly clash with USP_Tth_ β4-α4 loop residues Gly109 and Leu110 (identified by a *dotted red surface*). *E*, modeling of the ATP molecule (from the USP_Tth_:ATP structure) in the binding pocket of USP_Sa_ (superimposed onto the USP_Tth_ structure) shows that the terminal phosphoryl group in ATP would also clash with the USP_Sa_ β4-α4 loop residue Asn341 (also identified by a *dotted red surface*). USP, universal stress protein; c-di-AMP, cyclic-di-adenosine monophosphate; USP_Sa_, *S. aureus* KdpD-USP.

The β4-α4 loop in USP_FG_ proteins mediates specific interactions with ATP ribose and triphosphate groups through residues in the consensus Walker A motif: G_n_-X2-G_n+2_-X9-G _n+12_-(S/T), where n is the position of the first Gly in the motif (Figs. 4*C* and 5*C*) (46). The main chain amino group of the first Gly (G_n_) in this motif hydrogen bonds to the 2′ and 3′ hydroxyl groups of ribose in ATP. The second Gly (G_n+2_) and the Ser and Thr residues interact with the second β-phosphoryl and γ-phosphoryl groups, respectively (Fig. 5*C*). The α-phosphoryl group is generally hydrogen-bonded to the main chain amino group of the residue next to Ser and Thr residues in the motif (46). Consistent with the absence of β-phosphoryl and γ-phosphoryl groups in c-di-AMP, the β4-α4 loop regions in the USP_Sa_ and KdpD_CDA_ proteins lack most of the Walker A motif residues. However, the first Gly in the Walker A motif (G_n_) is fully conserved in USP_Sa_ (Gly329) and other KdpD_CDA_ proteins (Figs. 4*F* and S2*B*). USP_Sa_ Gly329, like G_n_ in ATP-binding USP_FG_ proteins, such as the G106 found in *Thermus thermophiles* (USP_Tth_ described in Fig. 5*C*), uses its main chain primary amino group to make polar contact with the 2′-hydroxyl and the 3′-oxygen of the inner ribose in c-di-AMP (Fig. 5*B*).

Interestingly, a structural comparison of USP_Sa_ proteins with USP_ATP_ proteins showed that the β4-α4 loop in USP_Sa_ adopts a conformation (hereafter referred to as a *CDA-bound conformation*) that is significantly different from the conformation in USP_ATP_ proteins (hereafter referred to as an *ATP-bound conformation*) (Fig. 5*A*). The different locations of the residues in each of these conformations seem to physically preclude the binding of a noncognate ligand in the active site. More specifically, in the ATP-bound conformation, the main chain of G_n+2_ and the side chain of the following residue in the Walker A motif of USP_ATP_ proteins (*e.g.,* Gly109 and Leu110 in USP_Tth_, which is shown in Fig. 5*D*) would sterically clash with the outer adenosine in c-di-AMP. Conversely, in the CDA-bound conformation, USP_Sa_ Asn341 would physically clash with the γ-phosphoryl group of ATP, potentially abrogating binding to ATP (Fig. 5*E*). In the CDA-bound conformation, two of the USP_Sa_ β4-α4 loop residues, Leu334 and His331, interact with c-di-AMP (Fig. 4, *A* and *D*). The His331-containing side chain makes a water-mediated hydrogen bond with the 2′ hydroxyl in the outer ribose (Figs. 4, *D* and *F*, and S2*B*), and the mutation of His331 to Ala results in ∼60% loss of relative c-di-AMP and AMP binding affinities (Fig. 4*E*). However, His331 is not conserved in KdpD_CDA_ proteins, and its role in c-di-AMP binding may be unique to USP_Sa_ (Fig. 4*F*). The other USP_Sa_:c-di-AMP interface residue in the α4-β4 loop, Leu344, is fairly conserved as Leu or Ile in KdpD_CDA_ proteins (Fig. 4*F*) and interacts with the inner AMP through (i) a main chain amino group–mediated hydrogen bond to one of the phosphoryl oxygens and (ii) through side chain–mediated hydrophobic interactions with the inner adenine (Figs. 4*A* and 5*B*). An L344A mutation significantly decreased the binding affinity of USP_Sa_ with c-di-AMP (∼10% relative affinity) and with AMP (40% relative affinity) (Fig. 4*E* and Table S1). The slightly lesser loss of affinity for AMP over c-di-AMP was unexpected, because Leu344 interacts only with the inner AMP in c-di-AMP, which is comparable to AMP. This may be because of a small side chain–induced increase in the flexibility of the β4-α4 loop, which could alter the CDA-bound conformation of this loop. The presence of another small side chain residue, Gly329, at the other end of this loop further supports this idea. The loop in its altered conformation may either better accommodate AMP and/or may disrupt the binding of c-di-AMP by inhibiting the outer ribose-specific interaction of His331 mentioned above. In summary, remodeling of the α4-β4 loop in USP_Sa_ seems to ensure proper positioning of Leu334 and His331 for interaction with c-di-AMP.

### Determinants of c-di-AMP specificity in USP_Sa_

To determine the molecular basis of c-di-AMP specificity in USP domains from KdpD_CDA_ proteins, we investigated the USPSa:c-di-AMP structural interface for USP_Sa_ contacts with the outer AMP moiety of c-di-AMP (absent in ATP and AMP). The significance of these contacts was examined by mutational analyses of the concerned USP_Sa_ residues to evaluate their c-di-AMP-binding and AMP-binding abilities. Among these USP_Sa_ residues, the first two, namely Ser244 and Ser246, comprise a part of the β1-α1 loop; the next two, Tyr248 and Asn249, reside at the beginning of the α1 helix; and the last four, Lys277, Arg279, Gln280, and Tyr281, are present in the β2-α2 loop (Fig. 4, *A* and *D*). All of these residues, except Ser244 and Tyr281, interact exclusively with the outer AMP moiety of c-di-AMP (Fig. 4, *A* and *D*).

Ser244 in the β1-α1 loop interacts with both the inner and outer AMP moieties of c-di-AMP, by making (i) hydrophobic interactions with the inner adenine and the inner ribosyl oxygen, as well as by forming (ii) a hydrogen bond with the phosphoryl oxygen of the outer AMP (Fig. 4, *A* and *D*). Consistent with this, an S244A mutant showed complete loss of c-di-AMP binding in our MST analysis (Fig. 4*E* and Table S1), which is an observation corroborated by a previous finding in a differential radial capillary action of ligand assay (56). Interestingly, the USP_Sa_ S244A mutant did not bind AMP *in vitro*, indicating a role for Ser244-mediated hydrophobic interactions with the inner AMP moiety of c-di-AMP (Fig. 4*E* and Table S1). While Ser244 is completely conserved in KdpD homologs (Figs. 4*F* and S2*B*), it is replaced by a conserved aspartate in all USP_FG_ proteins (Figs. 4*C* and S2*A*), where its side chain hydrophobically interacts with the adenine ring of ATP (Fig. 4*B*, and mentioned above). Further, our *in silico* Ser244 mutagenesis predicted that an aspartate at this position in USP_Sa_ would sterically clash with the outer adenosine in c-di-AMP (data not shown). We therefore hypothesized that a USP_Sa_-S244D mutation would reinstate hydrophobic interactions with AMP, while disrupting polar interactions with, and/or introducing steric clashes with, the outer AMP moiety of c-di-AMP. Indeed, an S244D mutant showed ∼50% relative binding affinity with AMP, whereas c-di-AMP binding was completely abolished (Fig. 4*E* and Table S1). Another residue in the β1-α1 loop, Ser246, which is conserved in many KdpD_CDA_ proteins (Figs. 4*F* and S2*B*), has a side chain that interfaces with the outer AMP through (i) hydrophobic interactions with the outer adenosine and (ii) a hydrogen bond with the outer phosphoryl oxygen (Fig. 4*D*). Moreover, an S246A mutant showed ∼40% relative binding affinity to both c-di-AMP and AMP (Fig. 4*E*).

Tyr248, located at the beginning of α1, interacts hydrophobically with the outer ribose through its side chain. Accordingly, the Y248A mutant showed a significant reduction in relative c-di-AMP binding (10% relative affinity), whereas its binding affinity to AMP was affected to a lesser extent (∼60% relative affinity) (Fig. 4*E* and Table S1). However, Tyr248 is only partially conserved in the KdpD_CDA_ homologs, with most of them containing a Ser or Thr at this position (Figs. 4*F* and S2*B*). The side chain of the next residue in the α1 helix, Asn249, forms polar interactions with one of the outer phosphoryl oxygens (Fig. 4*D*). Asn249 is completely conserved in most KdpD homologs (Figs. 4*F* and S2*B*), and an N249A mutant showed a significant reduction in c-di-AMP, whereas its binding affinity to AMP was not affected at all (Fig. 4*E* and Table S1). Furthermore, USP_ATP_ proteins contain a nonbulky residue (Ala, Ser, or Thr) at this position (Fig. 4*C*), suggesting an essential role of this residue that insures cyclic-dinucleotide specificity in USP_KdpD_ proteins.

Structural comparisons of USP_Sa_ and USP_FG_ proteins showed that the α2 helix in USP_Sa_ is shorter (>2 full turns) than in many of the USP_FG_ proteins (Fig. 4, *A* and *B*, and data not shown). This brings Tyr281 and potentially other unbuilt β2-α2 loop residues with extended side chains (Lys277, Arg279, and Gln280) closer to the nucleotide-binding pocket (Fig. 4, *A* and *D*). Tyr281 interacts with both the inner and the outer AMPs in c-di-AMP by (i) forming antiparallel sandwich-type π-stacking interactions with its terminal hydroxyl facing N7 and N9 of the outer adenine ring and by (ii) forming a water-mediated hydrogen bond with the inner phosphoryl oxygen. While we could not assess the binding energy contribution of the π-stacking interactions, because of the insolubility of our USP_Sa_-Y281A mutant, a more conservative, but soluble, USP_Sa_-Y281F mutant showed only ∼30% binding to c-di-AMP, as well as to AMP (Fig. 4*C* and Table S1), which underscores the interaction of the water-mediated polar interactions of Tyr281 with the inner phosphoryl group. Among the unbuilt β2-α2 loop residues, the relative c-di-AMP and AMP-binding affinities of both the K277A and the R279A mutants were reduced to ∼30% and ∼45%, respectively, whereas the Gln280A mutant showed relative c-di-AMP and AMP binding affinities of ∼60% and ∼95%, respectively (Fig. 4*E* and Table S1). While these β2-α2 loop residues contribute to c-di-AMP binding in USP_Sa_, they are not conserved in KdpD_CDA_ proteins (Figs. 4*F* and S2*B*), which indicates an altered c-di-AMP binding mode in the homologs.

In summary, our biochemical analyses identified eight residues (namely, Ala242, Leu344, Ser244, Tyr281, Ser246, His331, Lys277, and Arg279) that are important for binding to both c-di-AMP and AMP and three residues (Tyr248, Gln280, and Asn249) that are essential for binding to c-di-AMP (Fig. 4*E*).

### Sequence-based assignment of c-di-AMP binding activity in USP proteins

**Figure 6:**
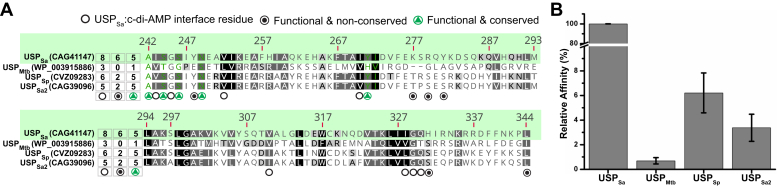
**Predicting c-di-AMPbinding activity in USP**_**Sa**_**homologs.***A*, sequence alignment of USP_Sa_, USP_Mtb_, USP_Sp_, and USP_Sa2_ (accession numbers shown in parentheses), with notations that summarize the number of USP_Sa_:c-di-AMP structural interface residues that are conserved in these homologs (at positions marked with *circles* below the alignment). *Circles* containing *green triangles* identify functionally important conserved interface residues; circles containing *black dots* identify functionally important nonconserved interface residues; and circles containing empty spaces identify functionally uncharacterized, nonconserved interface residues (see Fig. 4, *E* and *F*, and S2*B*). The numbers above the alignment identify residue positions in the USP_Sa_ domain within *S. aureus* KdpD (accession number CAG41147). The relative conservation of residues at each position is indicated by the shades of *gray* in the background, where the most conserved residues in the alignment are highlighted in *black*. To include all the KdpD_Sa_ homologs from Firmicutes and Proteobacteria that were previously compared in Figures 4*F* and S2*B* (and not just the four homologs that are compared in this figure) the background shading scheme (reflecting the conservation at each position) is based on all of the alignments in Figures 4*F* and S2*B*. *B*, shows relative binding affinities (%) of c-di-AMP (*gray bars*) for USP_Sa_, USP_Mtb_, USP_Sp_, and USP_Sa2_. The values of the dissociation constants (K_D_s) used for these measurements are summarized in Table S1. c-di-AMP, cyclic-di-adenosine monophosphate; USP, universal stress protein; USP_Sa_, *S. aureus* KdpD-USP.

Out of a total of 11 USP_Sa_ residues important for c-di-AMP binding, five were found to be conserved in >65% of the KdpD_CDA_ proteins (see circles with green triangles in Figs. 4, *C* and *F*, S2*B*, and 6*A*). This included two KdpD homologs, *L. monocytogenes* KdpD (accession number EDN8844575) and KdpD_Sa_ paralog (locus tag SAR0069) from *S. aureus* MRSA252, which were previously shown to bind c-di-AMP *in vitro* (Fig. S2) (56, 57). Therefore, a consensus motif from these five positions (A_242_/G/C)XS_244_XS_246_X2N_249_(Y_271_/F) was hypothesized to help predict the c-di-AMP-binding ability in KdpD_Firmicutes_. To test this hypothesis, we analyzed KdpD-USP domains from *Streptococcus pneumoniae* (named USP_Sp_) and *Mycobacterium tuberculosis* (named USP_Mtb_), where c-di-AMP signaling has been well characterized (27, 64, 65, 66). While USP_Sp_ conserves all five of these interfacial residues, USP_Mtb_ conserves only one (Figs. 6*A* and S2*B*). Indeed, the purified USP_Mtb_ exhibited only ∼0.7% relative c-di-AMP binding affinity (as compared with USP_Sa_) in the MST assay. USP_Sp_ and USP_Sa2_, on the other hand, showed higher relative binding affinities toward c-di-AMP (6.2% and 3.4%, respectively) (Fig. 4*B*). The lower binding affinities of USP_Sp_ and USP_Sa2_ relative to USP_Sa_ was consistent with their lack of the other four functionally important residues at the six nonconserved USP_Sa_:c-di-AMP interface positions (see circles filled with black dots in Figs. 4, *C* and *F* and 6*A*, and Table S1). Therefore, it is tempting to believe that the residue identities at these nonconserved positions in a USP protein, although not critical to binding, might still contribute to its c-di-AMP binding affinity. However, a more detailed analysis of these and other residues not tested in this study (empty circles in Fig. 4, *C* and *F*, and 6*A*) is required to make more generalized conclusions. Nonetheless, these residues' functional relevance in USP homologs for binding c-di-AMP further confirmed the validity of the observed USP_Sa_:c-di-AMP structural interface.

## Discussion

Despite a few functional studies, signal perception by the USP domain in the widespread KdpD family of HKs is poorly understood (33, 38, 39, 58, 67, 68, 69, 70). The discovery that the *S. aureus* KdpD USP_Sa_ is a c-di-AMP receptor, identified KdpD as a unique HK possessing a sensory domain dedicated to the sensing of cyclic di-nucleotides in bacteria (56) (Fig. 1*A*). There are other HK receptors (*e.g.,* the c-di-GMP-binding RavS HK of *Xanthomonas campestris* and the CckA HK of *Caulobacter crescentus*) that utilize their catalytic domains for binding cyclic di-nucleotides (71, 72). In this study, we determined the characteristic structural features of USP_Sa_, and we identified the principles governing ligand specificity in KdpD-USP domains from Firmicutes. An overall HUP domain topology (hereafter called HD-like) (73), formed by USP_Sa_ in the USP_Sa_:c-di-AMP structure, conserves a nucleotide-binding pocket comparable to standalone USP_FG_ proteins that bind ATP (Fig. 2) (46). The interactions of this pocket in USP_Sa_ with the inner adenine in c-di-AMP are very similar to the interactions of USP_FG_ proteins with the adenine in ATP (Fig. 4, *B* and *F*). It is worth noting that there is a significant contribution of Ala242 in this pocket in USP_Sa_ toward interactions with the inner adenine. Substituting a glycine for this alanine drastically increased USP_Sa_ binding affinity for both c-di-AMP and AMP (Fig. 4*E* and Table S1). Interestingly, all KdpD_CDA_ proteins and USP_ATP_ proteins contain a nonbulky residue at this position. In fact, a cyclic AMP-binding standalone USP protein from *M. tuberculosis* that naturally contains a Gly at this position had significantly reduced cyclic AMP binding affinity when this Gly was mutagenized to an Ala (53). Despite similarities in the overall structural fold and mode of engaging the inner adenine ring, USP_Sa_ contains several structural features that distinguish it from the standalone USP_FG_ proteins, and they regulate the substrate specificity, as discussed below.

### Structural basis of nucleotide specificity in USPs

Despite the significant structural similarity between USP_Sa_ and the standalone USP_FG_ proteins, these proteins exhibit preferential binding to c-di-AMP and ATP, respectively (56). While the absence of a Walker A motif in the β4-α4 loop of USP_Sa_ could alone accomplish the ATP-binding loss (46), this loop was found to adopt a conformation that is structurally distinct from its conformation in standalone USP_FG_ proteins (Fig. 5, *A–C*). This β4-α4 loop conformation not only precludes ATP binding by potential steric clashes with terminal phosphoryl groups (Fig. 5*E*) but it also positions functionally important β4-α4 loop residues (His331 and Leu334) for interaction with c-di-AMP (Fig. 5, *B* and *C*). Conversely, the conformation of this loop in USP_FG_ proteins seems to preclude c-di-AMP binding, because of the loop residues sterically clashing with the outer AMP in c-di-AMP (Fig. 5*D*). However, if the conformational change in the β4-α4 loop was the only way to distinguish c-di-AMP from ATP, AMP should not bind USP_Sa_ with an affinity significantly lower than that of c-di-AMP (Fig. 1*D*). In this context, it was perplexing as to how two AMP moieties (in c-di-AMP) are accommodated by a monomeric USP_Sa_, as opposed to one AMP moiety (in ATP) being accommodated by the dimeric standalone USP_FG_ proteins. The interactions of USP_Sa_ residues with the outer AMP in c-di-AMP underlie this nucleotide preference. More specifically, the interactions of residues in the β1-α1 loop (Ser244 and Ser246) and the β2-α2 loop (Tyr281, Lys277, Arg279 and Gln280) sandwich the outer adenine ring in an uncharacteristic *intermediate syn* conformation (Figs. 3 and 4, *D* and *E*). Also, the interactions of residues at the tip of α1 (Tyr248 and Asn249) with the outer ribose and the phosphoryl groups facilitate the specific binding of the outer AMP moiety to USP_Sa_. Of note are the interactions of the highly conserved Asn249 at the N-terminal tip of the α1 helix with the outer AMP (Fig. 4, *D–F*). By contrast, the inability of USP_ATP_ proteins to bind c-di-AMP could be due, at least in part, to (i) their longer (than USP_Sa_) α2 helix, which precludes an atypical inward-facing β2-α2 loop conformation, and its access to the outer adenine in c-di-AMP (Fig. 4, *A* and *B*) and (ii) their lack of conservation of the functionally important USP_Sa_ β1-α1 loop and the α1 residues (Ser244, Ser246, and Asn249).

### c-di-AMP binding activity in KdpD-USP domains

Despite the significant sequence divergence of USP domains in KdpD proteins (35), little can be predicted about their functional consequences. A sequence-based identification of c-di-AMP binding activity in USP proteins was not possible in the absence of a structure-function analysis of a KdpD-USP:c-di-AMP complex. Nevertheless, homologs of *S. aureus* KdpD in Firmicutes and Proteobacteria have previously been found to conserve the motif S_244_XS_246_-X20-F_267_T_268_A_269_XY_271_, where alanine substitutions of USP_Sa_-Ser244 and Tyr271 abrogated c-di-AMP binding *in vitro* (56). While our study confirmed the importance of Ser244 and Ser246 in this motif, the residues Phe267, Thr268, and Ala269 were not found at the USP_Sa_:c-di-AMP structural interface (Fig. 4). We therefore identified eight additional functionally important residues in USP_Sa_, two out of which, Ala242 and Asn249, are conserved in Firmicutes (Fig. 4, *E* and *F*). Based on the identified consensus binding motif (A/G/C)-X-S-X-S-X2-N-(Y/F), we were able to predict the c-di-AMP-binding ability of USP_Sa_ homologs from the human pathogens *M. tuberculosis* and *S. pneumoniae.* Consistent with this, proteobacteria that do not use c-di-AMP signaling (*e.g., E. coli*) lack this motif in KdpD proteins, further supporting the conclusions of our structural study.

### USP_Sa_ exhibits a unique nucleotide-binding mode in three-layered αβα sandwich proteins

The USP family of proteins contain a widespread three-layered αβα sandwich architecture, where a β-sheet layer is sandwiched between two α-helical layers (73). The most ubiquitous lineages possessing this architecture, namely HD-like Rossmann (74) and P-loop domain–like lineages (75), bind phosphorylated ribonucleoside ligands (called phospho-ligands below). The hallmark of these lineages are polar interactions of the phosphate and ribose moieties in the phosphor-ligands by glycine residues, and in some cases, serine residues from the β1-α1 loop and the N-terminal tip of the α1 helix (hereafter called the *α1-binding mode*, Fig. S3) (76). However, members of the HD-like lineage belonging to the USP and electron-transport flavoprotein families (ECOD F-groups 2005.1.1.145 and 2005.1.1.132, respectively), hereafter referred to as USP_ATP_ and ETF, instead use the β4-α4 loop and the N-terminal tip of the α4 helix (containing the Walker A motif, discussed above) for interactions with the phosphate and ribose moieties of ATP and cAMP (hereafter referred to as the *α4-binding mode*, Figs. 4*B* and 5*E*) (46, 53, 76). While the α4-binding mode exhibited by the USP_ATP_ and the ETF families sets them apart from other αβα sandwich proteins that exhibit the α1-binding mode, the evolutionary links between the α1-and the α4-binding modes are not known. Interestingly, USP_Sa_ seems to utilize both the α1-binding mode and the α4-binding mode to interact with the inner and outer AMP moieties in c-di-AMP (Figs. S3 and 4, *A* and *D*). More specifically, the interactions of the ribose and phosphate moieties of outer AMP with the conserved β1-α1 loop and the α1-tip residues discussed above (Ser244, Ser246, and Asn249) underlie the α1-binding mode. Furthermore, the interactions of β4-α4 loop residues (Gly329 and Leu344) with the inner AMP comprise a partial α4-binding mode (Figs. S3, 4*D* and 5, *B* and *C*). It is therefore possible that AMP adopts either an α4-binding mode or an α1-binding mode for interacting with USP_Sa_. This view is consistent with the fact that three of our USP_Sa_ mutants, L344A, S246A, and H331A, show unexpected differential binding effects toward AMP and c-di-AMP. More specifically, the alanine mutants of Ser246 and His331, which interact only with the outer AMP in the USP_Sa_:c-di-AMP structure, showed some AMP binding loss. Conversely, Leu344, which interacts only with the inner AMP, showed a greater binding loss toward c-di-AMP than AMP (Fig. 4, *A*, *D*, and *E*).

The “dual” mode of phospho-ligand binding observed in the USP_Sa_:c-di-AMP structure was not found in the proteins from the HD-like lineages, Rossmann-like lineages, and P-loop-domain-like lineages. This, along with the unique structural elements in USP_Sa,_ such as the shorter α2-helix, the inward-facing conformations of β4-α4 and β2-α2 loops, the lack of a Walker A motif, and the lack of a c-di-AMP-binding motif, support the idea that USP_Sa_-like c-di-AMP-binding domains from Firmicutes constitute a unique subfamily of the USPs.

This subfamily may represent an evolutionary link between USP_ATP_ proteins that utilize the α4-binding mode and other αβα-sandwich proteins utilizing an α1-binding mode (Rossmann-like lineages, P-loop-domain-like lineages, and most families in HD-like lineages) (Fig. 4, *A–C*) (7, 56). It will be interesting to see whether other USP-containing multidomain proteins, including transport proteins (such as Na^+^/H^+^ antiporters, Cl^−^ voltage channels, and amino acid permeases) (55, 77), and catalytic proteins (such as Ser/Thr protein kinases) (45, 46), exhibit similar structural features in their USP domains once their cognate ligands are found. Also, it will be interesting to elucidate whether the USP domains in these multidomain proteins also exist as monomers, like USP_Sa_ (Figs. 1*B* and S1), or whether they exist as dimers, like standalone USP_FG_ proteins (46).

The c-di-AMP-bound USP_Sa_ structure presented here completes the structural repertoire for all of the individual domains in the KdpDE TCS (41, 42, 43). However, it is yet to be determined how this TCS's function is regulated by the binding of c-di-AMP to KdpD in Firmicutes. In this context, the cellular accumulation of c-di-AMP has been shown to prevent the salt stress induced transcriptional up regulation of the *kdpFABC* operon in *S. aureus* (56). Consequently, the underlying mechanism may involve regulation of the KdpD and/or KdpE activities (34, 78). The N-terminal region of *E. coli* KdpD_Ec_ employs both such mechanisms: (i) the binding of ATP to the KdpD' domain regulates the relative autokinase and phosphatase activities of the KdpD_Ec_ HK domain (35, 39); and (ii) the interactions of the standalone USP-C protein with USP_Ec_ in KdpD_Ec_ scaffolds the KdpDE TCS with the target DNA (40). While our observed solubilization of the USP_Sa_ domain upon co-expression with *kdpE* in *E. coli* supports the latter model, a further characterization of a KdpD homolog from Firmicutes will be needed to determine the underlying regulatory mechanism.

## Experimental procedures

### Cloning and overexpression of USP domains

For structural and biochemical characterization of USP_Sa_ (accession number CAG41147, residues T213-N364), soluble preparations of His_6_-tagged and untagged USP_Sa_ were obtained by co-expressing the *usp*_Sa_ (*S. aureus* MRSA252 locus tag SAR2166) with the *S. aureus kdpE* gene (accession number SAR2167) in *E. coli.* For this, *usp*_*Sa*_ and full-length *kdpE* were first PCR amplified from *S. aureus* MRSA252 genomic DNA using Phusion High-Fidelity DNA polymerase (New England Biolabs) and primer pairs Untagged_USP_Sa_(WT)_F/Untagged_USP_Sa_(WT)_R and C-His-KdpE_F/C-His-KdpE_R, respectively (Table S3). PCR-amplified *usp*_*Sa*_ was then cloned into the *Nde*I and *EcoR*I site of a pBB75 vector using In-Fusion Cloning (Takara Bio USA), yielding the pBB(USP_Sa_) recombinant plasmid. The PCR-amplified *kdpE* was cloned into the *Nde*I and *Xho*I sites of pET21b to obtain the pET21(KdpE-His_6_) recombinant plasmid. The pBB(USP_Sa_) was then co-transformed with pET21(KdpE-His_6_) into chemically competent *E. coli* C41 (DE3) to co-express untagged USP_Sa_ and C-terminally hexa-histidine-tagged KdpE (KdpE-His_6_). For biochemical analyses, N-terminally His-tagged USP_Sa_ and untagged KdpE were cloned as follows. The *usp*_*Sa*_ and *kdpE* were PCR amplified using primer pairs N-His-USP_Sa_ (WT)_F/N-His-USP_Sa_ (WT)_R and Untagged_KdpE_F/Untagged_KdpE_R, respectively. The resulting *usp*_*Sa*_ amplicon was cloned into the *Pst*I and *Hind*III sites of pQLinkH (79), yielding pQLink(His_6_-USP_Sa_). The *kdpE* amplicon was cloned into *Nde*I and *EcoR*I sites of pBB75 to produce pBB75(KdpE). The pQLink(His_6_-USP_Sa_) and pBB75(KdpE) were co-transformed and expressed as above for His_6_-USP_Sa_ production. The mutations targeting the USP_Sa_:c-di-AMP interface were generated in the pQLink(His_6_-USP_Sa_) plasmid utilizing a Q5 site-directed mutagenesis kit (New England Biolabs), following the manufacturer's protocol.

For overexpression of the native and mutagenized USP domains, a single *E. coli* C41 (DE3) transformant colony containing two plasmids encoding USP_Sa_ and KdpE was grown in lysogeny broth supplemented with ampicillin (0.1 mg/ml) and kanamycin (0.05 mg/ml) to 0.6 OD_600_ at 37 °C and then induced with 0.25 mM Isopropyl ß-D-1-thiogalactopyranoside (IPTG). The culture was further grown at 15 °C for 16 h and harvested by centrifugation at 4000 rpm for 20 min.

Selenomethionine (SeMet)-derivatized USP_Sa_ domain (SeMet-USP_Sa_) was obtained by co-expression of pBB(USP_Sa_) and pET21(KdpE-His_6_) in L-methionine-free auto-inducible synthetic medium supplemented with 0.1 mg/ml ampicillin, 0.05 mg/ml kanamycin, and 125 μg/ml SeMet, as described previously (80). These cells were grown at 30 °C for 48 h, and harvested as described above.

For comparing the c-di-AMP binding abilities of different USP homologs from Firmicutes, the proteins were produced as N-terminal His-Sumo-tagged forms, without *kdpE* co-expression. For this, genes encoding the USP_Sa_ homologs, USP_Sa2_ (*S. aureus* MRSA252 KdpD with accession number CAG39096, residue range 225–374), USP_Sp_ (*S. pneumoniae* KdpD with accession number CVZ09283, residue range T208 to H359), and USP_Mtb_ (*M. tuberculosis* KdpD with accession number WP_003915886, residue range T216 to H378) were PCR amplified (using the primers identified with the prefix “N-SUMO-His” in Table S3), and the PCR amplicons were then cloned into the *Sap*I and *Xho*I sites of pTB146 (81). The expression of recombinant pTB146 plasmids containing USP_Sa2_, USP_Sp_, USP_Sa_, and USP_Mtb_ were performed as above, except that no KdpE was co-expressed, and kanamycin was omitted from the growth medium.

The cloning and mutagenesis of all recombinant plasmids used in this study were confirmed by Sanger sequencing carried out by Psomagen, Inc.

### Purification of USP domains

The bacterial cells expressing USP domains were resuspended in buffer A (50 mM potassium phosphate buffer, pH 8.0, and 300 mM NaCl) supplemented with a set of protease inhibitors (1 μg/ml each of aprotinin, leupeptin, and pepstatin and 10 μg/ml of phenylmethylsulfonyl fluoride) and 5 mM ß-mercaptoethanol. Cells were lysed using an Avestin Emulsiflex C3, and the lysate was subjected to centrifugation at 11,000 rpm for 1 h to remove cell debris. The supernatant was applied to a HisTrap Fast Flow column (GE Life Sciences) preequilibrated in buffer A. The column was washed with buffer B (50 mM potassium phosphate, pH 5.0, 300 mM NaCl, 5 mM MgCl_2_, 5 mM ATP) to remove impurities, and protein elution was performed using a linear gradient of buffer A and buffer C (50 mM potassium phosphate buffer, pH 8.0, 300 mM NaCl, and 0.1 M EDTA). USP and KdpE were eluted as separate peaks from the HisTrap Fast Flow column. The fractions containing USP were pooled, concentrated to 1 mM, and subjected to gel filtration chromatography using a Superdex 200 16/70 column preequilibrated with (i) buffer D (20 mM Tris-HCl, pH 8.0, 500 mM NaCl, 5 mM MgCl_2_, 5% glycerol, and 5 mM dithiothreitol) for untagged USP_Sa_ and SeMet-USP_Sa_ proteins and (ii) buffer A for His_6_-and His_6_-SUMO-tagged USP_Sa_ in its WT or mutant form. The USP_Sa_-containing peak fractions from the Superdex 200 column were concentrated to 1.0 to 1.5 mM with an Amicon Ultra-10 kDa cut-off centrifugal filter (Millipore) and stored at −80 °C.

### Sedimentation velocity analytical ultracentrifugation

SV-AUC experiments were performed at 20 °C with an XL-A Analytical Ultracentrifuge (Beckman Coulter) and a TiAn60 rotor with two-channel Epon charcoal-filled centerpieces and quartz windows. Protein samples were dissolved in buffer E (20 mM Tris-HCl, pH 8.0, 500 mM NaCl, and 5 mM MgCl_2_) in the presence or absence of 150 μM c-di-AMP. Complete sedimentation-velocity profiles were recorded every 30 s at 40,000 rpm and 280 nm.

The data were fitted using the c(*s*) distribution model of the Lamm equation, as implemented in SEDFIT (82). After optimizing the meniscus position and the fitting limits, sedimentation coefficients (*s*) and frictional ratios (*f/f*_*0*_) were determined by iterative least-squares fitting of the Lamm equation, with all root-mean-square deviations being less than 0.01. Final *s* values were converted to *s*_20,w_. The partial specific volume ($\bar{\nu }$ = 0.74942 ml/g), solvent density (ρ = 1.02280 g/ml), and viscosity (η = 0.01075 P) were derived from the chemical composition with the aid of sedimentation utility software (SEDNTERP) (83). The figures were prepared using the GUSSI program (84). Calculated hydrodynamic properties for monomeric and type I dimeric models of USP_Sa_ were determined using the WinHydroPro program (85). The dimeric model (type I) for WinHydroPro analysis was obtained through the use of the symmetry operation [X, Y, −Z+1] for the monomeric USP_Sa_:c-di-AMP complex in the asymmetric unit (86).

### Thermal shift assays

Solutions of 10 μl of 400 μM USP_Sa_, 6 μl of 300 x SYPRO Orange Protein Gel Stain (Invitrogen), and different concentrations (0–300 μM) of c-di-AMP in buffer F (20 mM Tris-HCl, pH 8.0, 500 mM NaCl, 5 mM MgCl_2_, and 5% glycerol) were added to the wells of a 96-well thin-wall PCR plate (Bio-Rad Laboratories). The plates were then sealed with Microseal “B” Seal Seals (Bio-Rad Laboratories) and heated in a CFX96 real-time PCR system (Bio-Rad Laboratories) from 25 °C to 99 °C in increments of 0.5 °C. Fluorescence changes in the plate wells were monitored simultaneously with a charge-coupled device camera, utilizing excitation and emission wavelengths of 497 nm and 520 nm, respectively.

### Crystallization, data collection, and structure determination

In a high-throughput crystallization screen, we identified a condition that exclusively yielded USP_Sa_ crystals in the presence of c-di-AMP. This crystallization condition was optimized to obtain crystals growing up to 300 × 200 × 50 μm in size, and diffraction data were collected at 2.3 Å resolution. Straightforward approaches to obtain phases for the USP_Sa_:c-di-AMP structure by molecular replacement with known USP homolog structures were unsuccessful. We therefore overexpressed and purified selenomethionyl-derivatized USP_Sa_ (SeMet-USP_Sa_) and determined phases for the USP_Sa_:c-di-AMP complex utilizing single-wavelength anomalous dispersion.

For crystal growth, the purified USP_Sa_ or SeMet-USP_Sa_ proteins were concentrated to 200 μM in buffer D and crystallized in the presence of a 10-fold molar excess of c-di-AMP (BIOLOG Life Science Institute). Crystals were formed in a 1:1 mixture of USP_Sa_:c-di-AMP (200 μM USP_Sa_ and 2 mM c-di-AMP in 20 mM Tris-HCl, pH 8.0, 500 mM NaCl, 5 mM MgCl_2_, 5% glycerol, and 5 mM dithiothreitol) and well solution (100 mM Tris-HCl, pH 8.5, 0.15 M Li_2_SO_4_, and 21% polyethylene glycol 3350) using a hanging-drop vapor-diffusion method at 20 °C. The crystals were soaked and cryoprotected for ∼15 s in the well solution supplemented with 10% glycerol and then flash-frozen in liquid nitrogen.

Diffraction data from nitrogen-cooled SeMet-USP_Sa_:c-di-AMP (Se-USP_Sa_-CDA) and USP_Sa_:c-di-AMP (USP_Sa_-CDA) crystals were obtained at the Advanced Light Source beamlines 5.0.2 and 5.0.1, respectively, at the Lawrence Berkeley National Laboratory in Berkeley, CA. The Se-USP_Sa_-CDA crystal diffracted up to 2.51 Å, whereas the USP-CDA crystal diffracted up to 2.3 Å. All of the diffraction data were indexed, integrated, and scaled, utilizing the HKL-2000 program package (87). The USP_Sa_:c-di-AMP crystal structure was determined by the single-wavelength anomalous dispersion method, using crystals of SeMet-USP_Sa_ bound to c-di-AMP that were isomorphous to the native USP_Sa_:c-di-AMP crystals. PHENIX (AutoSol) was used to locate five selenium positions, calculate phases, and generate an initial model at 2.51 Å resolution (88). This model was then refined against 2.3 Å native data, utilizing the PHENIX refinement. Iterative rounds of manual model building in Coot (89) and refinement in PHENIX generated the final model with R_work_ = 20.8 and R_free_ = 24.9. The electron density map showed good agreement with the modeled polypeptide chain, although no density was obtained for residues 213 to 235, 277 to 279, and 364. The bound c-di-AMP molecule was identified by inspection of the F_O_-F_C_ electron density map and was manually modeled. The PyMOL molecular visualization system (Version 2.3.2) was used to perform all structural analyses of the groups' distance and nature and to generate structural illustrations.

### USP_Sa_ structural and sequence comparisons

For comparisons with KdpD_Firmicutes_, the amino acid sequences of 27 KdpD_Sa_ homologs from representative Firmicute and Proteobacterial species co-harboring genes encoding a protein containing a diadenylate cyclase domain were aligned with USP_Sa_ using Blosum62 matrix in Geneious Prime software (Biomatters Ltd). The homologs from representative species were first identified with a BLAST search of the USP_Sa_ sequence, and the genomes of the corresponding hits were then confirmed to be containing a c-di-AMP cyclase with homology to either *S. aureus* DacA or *Bacillus subtilis* DisA by another BLAST search. The 27 KdpD_Sa_ homologs that were identified are referred to as KdpD_CDA_ throughout the text.

For structure-guided sequence alignment of ATP-binding USP_FG_ proteins with USP_Sa_, three-dimensional structures of eight structurally characterized ATP-bound USPs (PDB IDs: 1MJH, 5AHW, 3S3T, 3FDX, 2JAX, and 3HGM), which are referred to as USP_ATP_ throughout the text, were aligned against the USP_Sa_ model in the USP_Sa_:c-di-AMP structure, using the PROMALS3D multiple sequence and structure alignment tool (90). The exported sequence alignments were then illustrated with Geneious Prime software.

### MicroScale Thermophoresis

The ligand-binding specificity of USP_Sa_, the role of USP_Sa_ residues in c-di-AMP binding, and the c-di-AMP binding affinity of USP homologs were biochemically determined by MST, using purified WT and mutant proteins. To compare the ligand-binding specificity between c-di-AMP and ATP, we used AMP as a proxy for ATP, because both AMP and ATP bind to USP_Sa_ with comparable affinities (Fig. 1*C* and Table S1), though ATP tends to get hydrolyzed in solution. For these MST experiments, WT and active-site mutants of His_6_-USP_Sa_ were first fluorescently labeled using a RED-Tris NTA Monolith His-Tag labeling kit (Nanotemper, Technologies, Inc). For labeling, the proteins were diluted to a concentration of 800 nM in binding buffer (50 mM potassium phosphate, pH 8.0, 800 mM NaCl, and 0.05% Tween 20), mixed with an equal volume of RED-Tris-NTA dye (80 nM), and incubated on ice in the dark for 30 min. The stock solutions of ligands (c-di-AMP, c-di-GMP, ATP, AMP, c-AMP) were serially diluted with the binding buffer in a 1:1 ratio during each dilution step. The labeled proteins and the ligand dilutions were then mixed in a 1:1 ratio and incubated on ice in the dark for 30 min.

The samples were then loaded into Monolith standard-treated capillaries in triplicate, and the changes in fluorescence were measured with 40% LED power and 20% IR-laser power for an on-time of 20-s at 23 °C. To determine the binding affinity, the ligand-dependent changes in the intensity of the initial fluorescence, or the thermophoretic mobility, were analyzed according to the law of mass action in a standard fitting mode of MO.Affinity analysis software (version 2.3). To exclude the possibility of nonspecific binding of the ligand to the His_6_-tag (on USP_Sa_) or ligand-induced USP adsorption to labware or due to aggregation, we performed His_6_-peptide and EDTA tests, respectively (91). In the His_6_-peptide test, a control His_6_-peptide was used instead of USP in the binding assay described above, and if no change in initial fluorescence occurred, this confirmed the USP-specific binding of ligands. In the EDTA test, the binding buffer additionally contained 50 mM EDTA to disrupt the interaction between His_6_-USP and the RED-Tris-NTA dye. The complete loss of the ligand-induced initial fluorescence change in this assay confirmed that this change was neither because of protein aggregation nor because of protein adsorption to labware.

To facilitate comparisons of nucleotide-binding affinities in Figures 1*C*, 4, and 6, *B* and *C*, all of the K_D_ values in Table S1 were first converted into association constants (K_a_) using the equation K_a_ = 1/K_D_. The K_a_ values of WT USP_Sa_ for c-di-AMP or AMP were set to 100% relative affinity, and the binding affinities of the other nucleotides (Fig. 1*C*), or USP_Sa_ mutants (Fig. 4*E*), or USP homologs (Fig. 6, *B* and *C*) were depicted as percent values compared with the values of the WT USP_Sa_.

## Data availability

Coordinates and structure factors for the USP_Sa_:c-di-AMP structure have been deposited in the RCSB Protein Data Bank (http://www.rcsb.org) with the accession code 7JI4. Strains and plasmids are described in this manuscript, and the raw data for the binding analyses in Figures 5 and Table S3 are available upon request.

## Supporting information

This article contains supporting information (56, 76).

## Conflict of interest

The authors declare that they have no conflicts of interest with the contents of this article.

## Supporting information

Figures S1–S3 and Tables S1–S4

All non-standard abbreviations are listed in Table S4.
