# Supplementary material for: Structural basis of KdpD histidine kinase binding to the second messenger c-di-AMP
Source: J Biol Chem. 2021 May 11;296:100771. doi: 10.1016/j.jbc.2021.100771 (PMC8214093; doi:10.1016/j.jbc.2021.100771)
Supplement: Figures S1–S3 and Tables S1–S4 [file mmc1.pdf]

# SUPPORTING MATERIAL

## Structure-function analysis of c-di-AMP binding to KdpD histidine kinase reveals a novel ligand binding mode in the USP family of proteins

**Anirudha Dutta, Mona Batish, and Vijay Parashar***

*Department of Medical and Molecular Sciences, 15 Innovation Way,*

*University of Delaware, Newark, DE 19711*

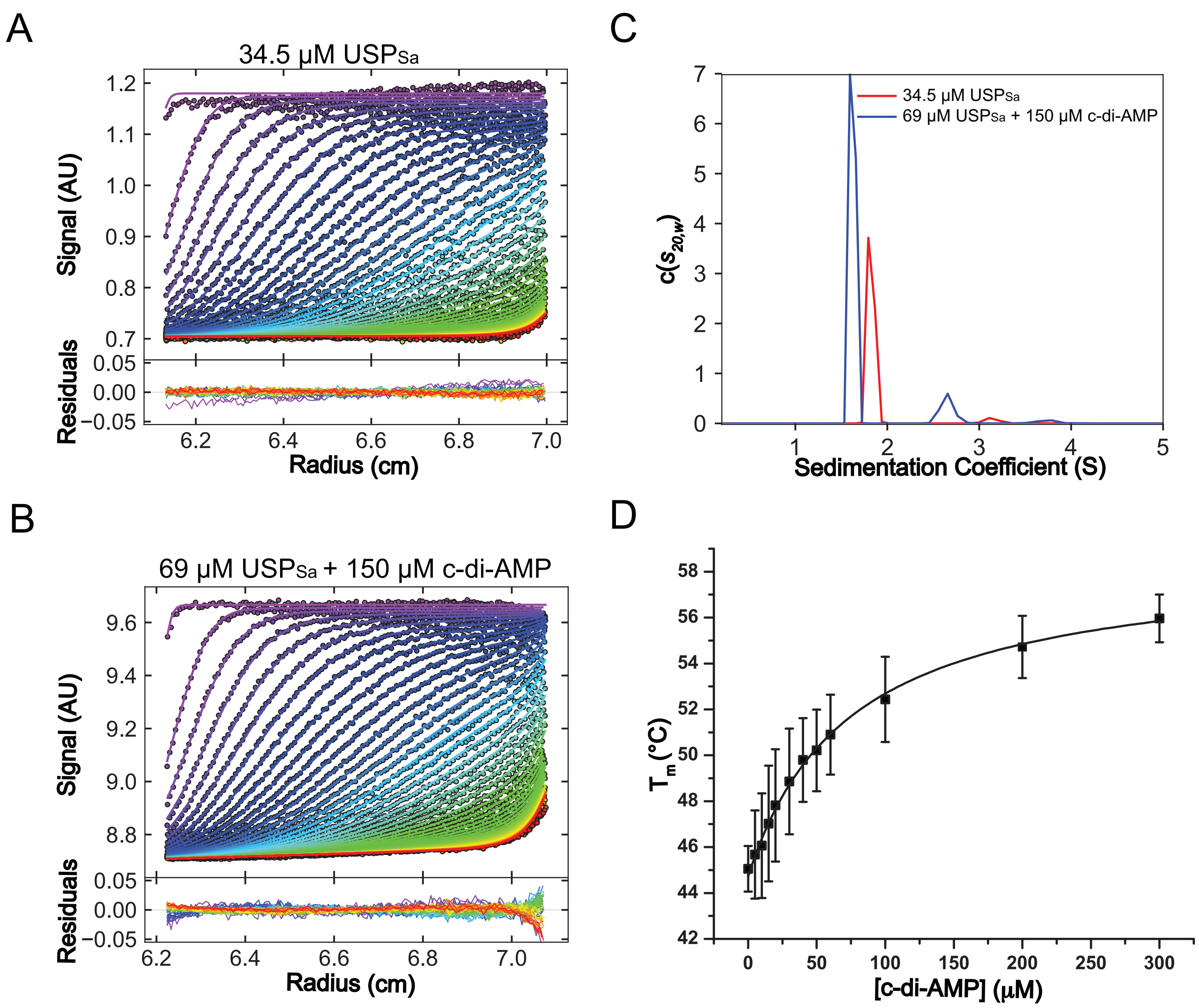


**Figure S1. C-di-AMP binding stabilizes USPSa in a monomeric form. *A* and *B*,** Representative absorbance data (colored circles) for sedimentation boundaries in SV-AUC are shown in the upper panel for 34.5 µM USPSa and in the lower panel for 69 µM USPSa, with 150 µM c-di-AMP as a function of radial position and time. Solid lines are the fits to the Lamm equation, as performed in SEDFIT. In the respective lower panels, the residuals of these fits are shown. ***C,*** c(S) distributions derived from the fitting of the Lamm equation for USPSa (red), and for USPSa with c-di-AMP (blue). ***D,*** Effect of c-di-AMP concentration on the thermal stability of USPSa, using the ThermoFluor assay.

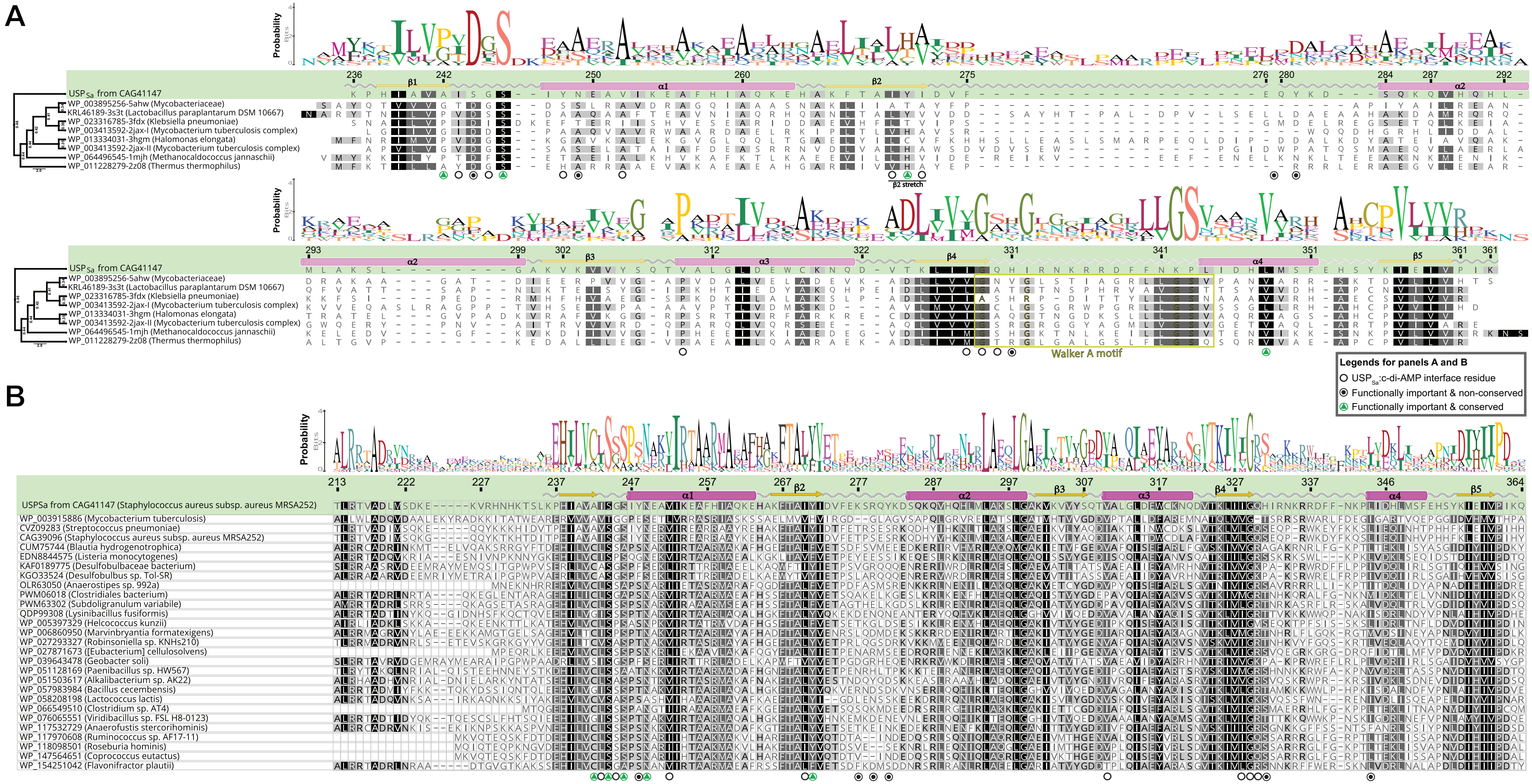


**Figure S2. Sequence alignments of USP$_{Sa}$ with other USPs.** ***A,*** Structure-guided alignment of ATP-binding USP$_{FG}$ proteins with respect to USP$_{Sa}$. The labels for each protein indicate its Protein Data Bank ID and its bacterial source. The *M. tuberculosis* UspE-type protein (Protein Data Base ID 2JAX) contains two tandemly arranged USP domains separated at residue 150, and is therefore shown as two subjects labeled “2jax-I” and “2jax-II” in this alignment. ***B,*** Sequence alignment of USP domains from KdpD$_{CDA}$ proteins (see Experimental Procedures for description). Each domain is identified by its accession number and bacterial source. The sequence alignments in panels A and B, and the neighbor-joining tree generation for panel A were performed using Geneious Prime software (https://www.geneious.com), and were edited with Adobe Illustrator (version 24.3). In panels A and B**,** the USP$_{Sa}$ residues at the USP$_{Sa}$:c-di-AMP structural interface are identified by circles below the alignment. Circles filled with green triangles identify residues that are conserved amongst the homologs (>65% similarity), and that showed significant loss or gain of function in the c-di-AMP binding assay (see panel C and (1)); circles filled with black dots identify

residues that are not conserved, but showed significant loss of function in the c-di-AMP binding assay; and empty circles show $USP_{Sa}$:c-di-AMP structural interface residues that were not subjected to mutagenesis and functional analysis. The conservation of residues at each position is depicted by the size of the letters in the sequence logo, where the most conserved residues are highlighted by a larger sized letter and a by a black background. Logo letters colored blue, green, red, and black indicate basic, polar, acidic, and hydrophobic residues, respectively. The numbering is based on the residue positions of the $USP_{Sa}$ domain within *S. aureus* KdpD (accession number CAG41147). The secondary structure elements are derived from the $USP_{Sa}$:c-di-AMP structure (in which α-helices are shown as magenta cylinders, and β-sheets are shown as yellow arrows).

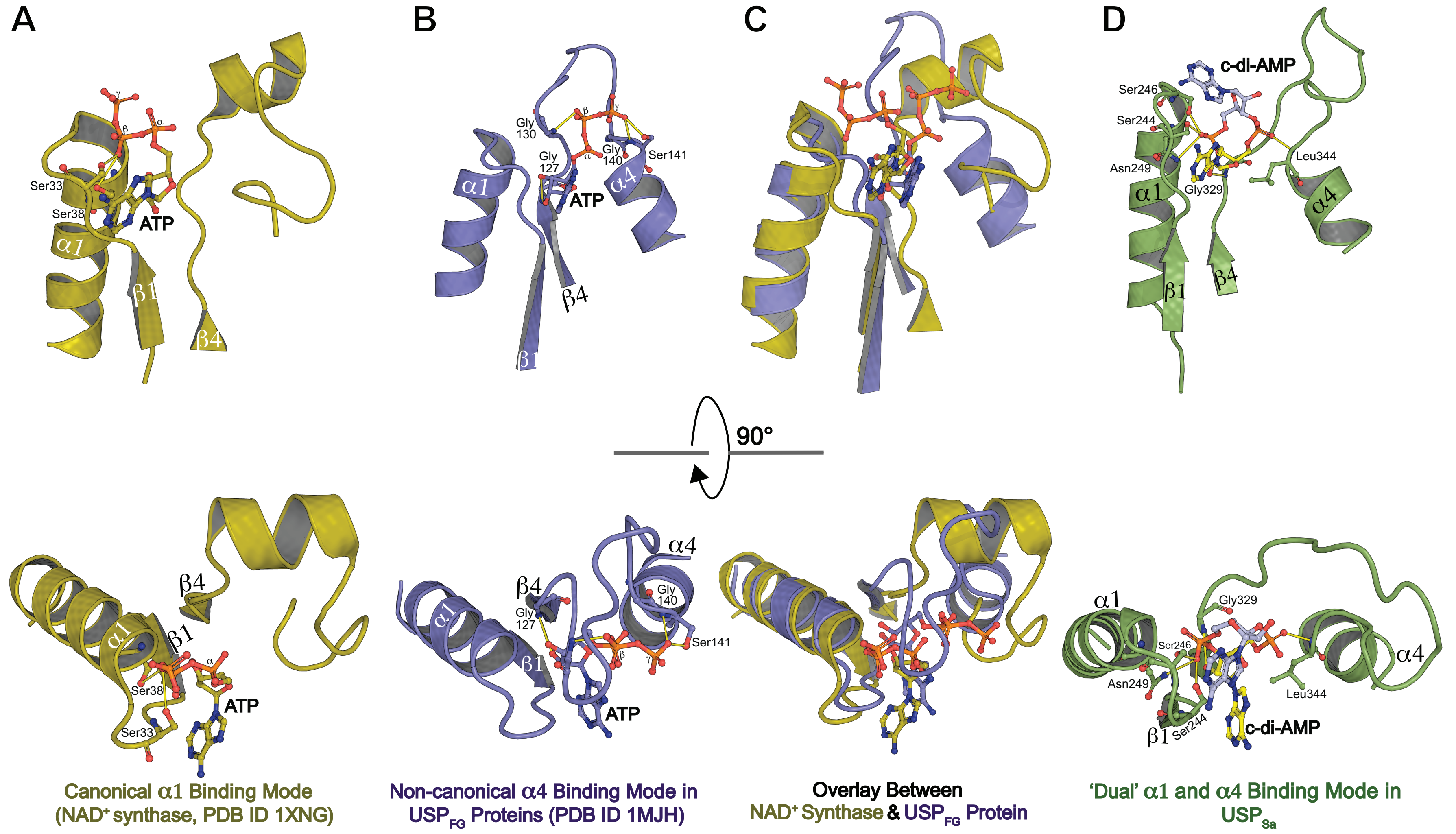


**Figure S3. USP$_{Sa}$ utilizes a dual phospho-ligand binding mode for interactions with the inner and outer AMPs in c-di-AMP.** ***A,*** Canonical α1-phospho-ligand-binding mode in the NAD$^{+}$ synthase family (ECOD F-group 2005.1.1.13; shown as a yellow cartoon for Protein Data Bank ID 1XNG). This mode is also conserved in Rossmann-like lineages (ECOD X-group 2003.1), and in P-loop-domain-like (ECOD X-group 2004.1) lineages (2). ***B,*** The α4-phospho-ligand binding mode in standalone ATP-binding and in cAMP-binding members of the USP$_{FG}$ family (ECOD F-group 2005.1.1.145; shown as a purple cartoon for Protein Data Bank ID 1MJH, which is a model USP$_{FG}$). ***C,*** Overlay of 1XNG and 1MJH from panels A and B, enabling a comparison of the orientations of their ATP terminal phosphoryl groups in the α1- binding mode and in the α4-binding mode, respectively. ***D,*** Illustration of the outer and inner AMP moieties in c-di-AMP interacting with USP$_{Sa}$ in the "dual" mode, which involves aspects of both the α1-binding mode and the α4- binding mode. The top and bottom rows of

each panel provide both a side view and a top view of each protein model.  For clarity, only α1, α4, β1, β4, and their connecting loops, are used for these structural alignments.

**Table S1: Dissociation constants ($K_D$s) of USP proteins for nucleotides tested in this study**

| Protein | Dissociation Constant ($K_D$) ± Standard Deviation | | | | | |
|---|---|---|---|---|---|---|
| | c-di-AMP (µM) | AMP (mM) | ATP (mM) | c-AMP (mM) | c-di-GMP | c-tri-AMP |
| $USP_{Sa}$-WT | 0.53 ±0.06 | 2.65 ±1.16 | 5.15 ±0.07 | 10.00 ±0.71 | No Binding Observed | No Binding Observed |
| $USP_{Sa}$-A242G | 0.01 ±0.010 | 0.14 ±0.01 | Not Measured | | | |
| $USP_{Sa}$-S244A | No Binding Observed | No Binding Observed | Not Measured | | | |
| $USP_{Sa}$-S244D | No Binding Observed | 4.06 ±1.94 | Not Measured | | | |
| $USP_{Sa}$-S246A | 1.30 ±0.19 | 7.25 ±0.21 | Not Measured | | | |
| $USP_{Sa}$-Y248A | 4.22 ±0.11 | 5.40 ±3.81 | Not Measured | | | |
| $USP_{Sa}$-N249A | 7.84 ±0.34 | 5.17 ±3.42 | Not Measured | | | |
| $USP_{Sa}$-Q280A | 0.87 ±0.06 | 1.82 ±0.25 | Not Measured | | | |
| $USP_{Sa}$-Y281F | 1.79 ±0.84 | 5.15 ±0.35 | Not Measured | | | |
| $USP_{Sa}$-K282A | 0.60 ±0.24 | 1.45 ±0.64 | Not Measured | | | |
| $USP_{Sa}$-H331A | 2.37 ±1.32 | 4.13 ±0.11 | Not Measured | | | |
| $USP_{Sa}$-L344A | 8.84 ±1.09 | 4.14 ±0.90 | Not Measured | | | |
| $USP_{Sa}$-V310A | 1.71 ±0.42 | 2.65 ±1.16 | Not Measured | | | |
| $USP_{Sa}$-K277A | 10.14 ±0.24* | 50.65 ±4.03* | Not Measured | | | |
| $USP_{Sa}$-R279A | 8.67 ±0.75* | 14.36 ±0.95* | Not Measured | | | |
| $USP_{Sa}$-WT | 3.13 ±0.75* | 20.74 ±4.40* | Not Measured | | | |
| $USP_{Sa2}$-WT | 124.95 ±15.01* | Not Measured | Not Measured | | | |
| $USP_{Sp}$-WT | 67.52 ±12.96* | Not Measured | Not Measured | | | |
| $USP_{Mtb}$-WT | 665.93 ±168.22* | Not Measured | Not Measured | | | |

*Measured using change in thermophoretic mobility in a microscale thermophoresis experiment. All other measurements made using changes in initial fluorescence, where nonspecific interactions and USP adsorption to labware /aggregation were excluded based on $His_6$-peptide and EDTA tests.

**Table S2. Calculated properties of $USP_{Sa}$ structural models versus properties determined by sedimentation velocity analysis**

| Sample | Species | Theoretical $s_{20,w}$ (Structural Models) | Experimental $s_{20,w}$ (Sedimentation Velocity) | Theoretical MW (kD) | Calculated MW ($M_f$) (kD) |
|---|---|---|---|---|---|
| $USP_{Sa}$ | Monomer | - | 1.9 | 17.5 | 20.1 |
| | Dimer | - | 3.3 | 35.0 | 44.9 |
| $USP_{Sa}$ + c-di-AMP | Monomer | 1.7 | 1.8 | 17.5 | 19.1 |
| | Dimer | 2.6 | 2.9 | 35.0 | 40.4 |

**Table S3. Sequences of oligonucleotides used in this study**

| Primer/ Construct Name | Oligonucleotide sequence (5’ to 3’) |
| --- | --- |
| Untagged_USP$_{Sa}$(WT)_ F | GAAGGAGATATACATATGACACTCAGAACTGTCGCAG |
| Untagged_USP$_{Sa}$(WT)_R | TATCTAGAGCTCGAATTCTTAATAATGAATCGGTAC |
| N-His-USP$_{Sa}$ (WT)_ F | ATCACCATGGTGCTGCAGGCACGTTAAGAACAGTTG |
| N-His-USP$_{Sa}$ (WT)_ R | GCTCAGCTAATTAAGCTTTTATTGTTTGATTGGAACG |
| Untagged_KdpE_F | GGAGATATACATATGGTATTTACTAACGGTTTG |
| Untagged_KdpE_R | CTAGAGCTCGAATTCTTATTTCTCTTTCCACTG |
| C-His-KdpE_F | GAAGGAGATATACATATGCAATCTAAAATATTGATAATTG |
| C-His-KdpE_R | GTGGTGGTGGTGCTCGAGTTTCTCTTTCCACTGCAAC |
| N-His-USP$_{Sa}$ (K282A)_F | GCAGGCAATATGCAGATAGTCAAAAGC |
| N-His-USP$_{Sa}$ (K282A)_R | TTTTTTCGAATACATCTATATAAATAGCAG |
| N-His-USP$_{Sa}$ (Y281A)_F* | CGAAAAAAGCAGGCAAGCTAAAGATAGTCAAAAGC |
| N-His-USP$_{Sa}$ (Y281A)_R* | AATACATCTATATAAATAGCAGTGAATTTTG |
| N-His-USP$_{Sa}$ (Y248A)_F | GTGGGAGTATTGCTAATGAAGCAGTAATTAAAG |
| N-His-USP$_{Sa}$ (Y248A)_R | TAATTGCCACAGCAATATGAGGTTTGAGTG |
| N-His-USP$_{Sa}$ (N249A)_F | GGGAGTATTTATGCTGAAGCAGTAATTAAAG |
| N-His-USP$_{Sa}$ (N249A)_R | ACTAATTGCCACAGCAATATGAGGTTTGAG |
| N-His-USP$_{Sa}$ (H331A)_F | TTAATTATCGGACAAGCTATTAGAAATAAGCG |
| N-His-USP$_{Sa}$ (H331A)_R | TTTGGTTACATCTTGATTTTTACACCATTCG |
| N-His-USP$_{Sa}$ (L344A)_F | CTTTTTCAATAAACCTGCAATTGACCATTTAATG |
| N-His-USP$_{Sa}$ (L344A)_R | TCTCGCCGCTTATTTCTAATATGTTGTCCG |
| N-His-USP$_{Sa}$ (Y271A)_F* | CAAAATTCACTGCTATTGCTATAGATGTATTCG |
| N-His-USP$_{Sa}$ (Y271A)_R* | CATGTTCTTTTTGAGCAATATGGAATGCC |
| N-His-USP$_{Sa}$ (I328A)_F* | GTAACCAAATTAATTGCCGGACAACATATTAG |
| N-His-USP$_{Sa}$ (I328A)_R* | ATCTTGATTTTTACACCATTCGTCTAATCC |
| N-His-USP$_{Sa}$ (S244D)_F | GCTGTGGCAATTGATGGGAGTATTTATAATGAAG |
| N-His-USP$_{Sa}$ (S244D)_R | AATATGAGGTTTGAGTGACGTTTTATGATTG |
| N-His-USP$_{Sa}$ (S246A)_F | GGCAATTAGTGGGGCTATTTATAATGAAGC |
| N-His-USP$_{Sa}$ (S246A)_R | ACAGCAATATGAGGTTTGAGTGACGTTTTATG |
| N-His-USP$_{Sa}$ (V310A)_F | GTTTATAGCCAAACCGCTGCATTAGGATTAG |
| N-His-USP$_{Sa}$ (V310A)_R | TACTTTTACTTTTGCTCCTAAAGATTTTGC |
| N-His-USP$_{Sa}$ (A242G)_F | CTCATATTGCTGTGGGAATTAGTGGGAG |
| N-His-USP$_{Sa}$ (A242G)_R | GTTTGAGTGACGTTTTATGATTGTGTCGGAC |

| | |
|---|---|
| N-His-USP$_{Sa}$ (S244A)_F | ATTGCTGTGGCAATTGCTGGGAGTATTTATAATG |
| N-His-USP$_{Sa}$ (S244A)_R | ATGAGGTTTGAGTGACGTTTTATGATTGTG |
| N-His-USP$_{Sa}$ (Q280A)_F | GTATTCGAAAAAAGCAGGGCATATAAAGATAGTC |
| N-His-USP$_{Sa}$ (Q280A)_R | ATCTATATAAATAGCAGTGAATTTTGCATGTTC |
| N-His-USP$_{Sa}$ (I270G)_F* | CACTGCTATTTATGGAGATGTATTCGAAAAAAG |
| N-His-USP$_{Sa}$ (I270G)_R* | AATTTTGCATGTTCTTTTTGAGCAATATGG |
| N-His-USP$_{Sa}$ (I270A)_F* | CACTGCTATTTATGCAGATGTATTCGAAAAAAG |
| N-His-USP$_{Sa}$ (I270A)_R* | AATTTTGCATGTTCTTTTTGAGCAATATGG |
| N-His-USP$_{Sa}$ (R279A)_F* | TTCGAAAAAAGCGCGCAATATAAAGATAG |
| N-His-USP$_{Sa}$ (R279A)_R* | TACATCTATATAAATAGCAGTGAATTTTGC |
| N-His-USP$_{Sa}$ (K277A)_F | GAAAAAAGCAGGCAATATGCAGATAGTCAAAAGC |
| N-His-USP$_{Sa}$ (K277A)_R | CAAAATTCACTGCTATTTATATAGATGTATTC |
| N-His-USP$_{Sa}$ (Y281F)_F | CGAAAAAAGCAGGCAATTTAAAGATAGTCAAAAGC |
| N-His-USP$_{Sa}$ (Y281F)_R | AATACATCTATATAAATAGCAGTGAATTTTG |
| N-His-SUMO--USP$_{Sa2}$ (WT)_F | GAACAGATTGGTGGTATTGAACGCTTATTAAATAT |
| N-His-SUMO--USP$_{Sa2}$ (WT)_R | GTCACCCGGGCTCGAGTTACAAATAAAAATAAAAA |
| N-His-SUMO--USP$_{Sp}$ (WT) | Gene block sequence:<br>AGAGAACAGATTGGTGGTACATTAAGAACTGTAGCGGATATCATGAGTCAGAAAGAACAGC<br>AGTATAAGAAGAAACATATAGATGTGACACCTCATATTGCTGTGGCTGTAAGTGGCAGTATT<br>TATAATGAACGCGTGATTCGCGAAGCAAGACGTGCTGCATATAGAGAACATGCAAAGTTTAC<br>GGCGATTTATATCGATACCTTTGAAACACGTTCTGAAAGTCGTAAGCAAGACCATTATATTCA<br>TAAAAATCTTACGCTAGCGAAGTCGTTGGGTGCTGAAATTAAAGTATTGTATGCACAAGATA<br>TTGCGAAGACGTTAATCAATTGGTGCGATAAATCCCTCGTGACCAAATTGGTGCTAGGCCAG<br>TCAGAACAACCTCGTTGGAAAGAGTACTTTAAGAAGTCACTCATTGAACAAATTAATCATACG<br>CCGCATCACTTTAAATTAGAGATTGTACCAATACATCACTAACTCGAGCCCGGGTGA |
| N- His-SUMO--USP$_{Mtb}$ (WT)_F | AGAGAACAGATTGGTGGTACGGGCAATCTGACCGCG |
| N-His-SUMO--USP$_{Mtb}$ (WT)_R | CAGTCACCCGGGCTCGAGTTAATGGGTGACCATGTG |

**Table S4: List of non-standard abbreviations**

| | |
|---|---|
| **PDB** | Protein Data Bank |
| **SV-AUC** | Sedimentation velocity analytical ultracentrifugation |
| **CDA** | 3', 5'-cyclic di-adenosine monophosphate (c-di-AMP) |
| **$KdpDE_{Ec}$** | KdpDE two-component system from *Escherichia coli* |
| **$KdpD_{Firmicutes}$** | KdpD histidine kinase homologs from Firmicutes |
| **$KdpD_{CDA}$** | Bioinformatically identified KdpD homologs that co-harbor a c-di-AMP cyclase |
| **$KdpD_{Sa}$** | KdpD histidine kinase homolog encoded by locus tag SAR0066 from *Staphylococcus aureus* MRSA252 |
| **$KdpD_{Ec}$** | KdpD histidine kinase homolog from *Escherichia coli* |
| **KdpD'** | KdpD domain in the N-terminal region of the KdpD histidine kinase |
| **$KdpE_{Ec}$** | KdpE response regulator from *Escherichia coli* |
| **GAF** | cGMP-specific phosphodiesterases, adenylyl cyclases, and bacterial transcription factor FhlA domain |
| **NTR** | N-terminal region in KdpD histidine kinase |
| **Standalone USP** | Independently translated universal stress protein domain |
| **$USP_{ATP}$** | Group of eight structurally characterized ATP-bound USPs (PDB IDs: 1MJH, 5AHW, 3S3T, 3FDX, 2JAX, and 3HGM) |
| **$USP_{Ec}$** | USP domain in the *Escherichia coli* KdpD |
| **$USP_{FG}$** | Walker A motif containing standalone USPs that bind adenosine triphosphate |
| **$USP_{KdpD}$** | USP domain in the N-terminal region of KdpD histidine kinases |
| **$USP_{Mj}$** | Standalone $USP_{FG}$ protein from *Methanocaldococcus jannaschii* (Protein Data Bank ID 1mjh) |
| **$USP_{Mtb}$** | USP domain (residue range T216 to H378) from *Mycobacterium tuberculosis* KdpD (accession number WP_003915886) |
| **$USP_{Sa}$** | USP residues T213-N364 (accession number CAG41147) encoded by *Staphylococcus aureus* MRSA252 locus tag SAR2166 |
| **$USP_{Sa2}$** | USP domain (residue range 225 to 374) from *Staphylococcus aureus* MRSA252 KdpD2 (accession number CAG39096) |
| **$USP_{Sp}$** | USP domain (residue range T208 to H359) from *Streptococcus pneumoniae* KdpD (accession number CVZ09283) |
| **$USP_{Tth}$** | Standalone $USP_{FG}$ protein from *Thermus thermophilus* HB8 (Protein Data Bank ID 2Z08) |

## Supplemantary References


1. Moscoso, J. A., Schramke, H., Zhang, Y., Tosi, T., Dehbi, A., Jung, K., and Gründling, A. (2016) Binding of Cyclic Di-AMP to the Staphylococcus aureus Sensor Kinase KdpD Occurs via the Universal Stress Protein Domain and Downregulates the Expression of the Kdp Potassium Transporter. *Journal of Bacteriology* **198**, 98-110
2. Longo, L. M., Jablonska, J., Vyas, P., Kanade, M., Kolodny, R., Ben-Tal, N., and Tawfik, D. S. (2020) On the emergence of P-Loop NTPase and Rossmann enzymes from a Beta-Alpha-Beta ancestral fragment. *Elife* **9**
